# Deltex1 antagonizes HIF-1α and sustains the stability of regulatory T cells *in vivo*

**DOI:** 10.1038/ncomms7353

**Published:** 2015-02-19

**Authors:** Huey-Wen Hsiao, Tzu-Sheng Hsu, Wen-Hsien Liu, Wan-Chen Hsieh, Ting-Fang Chou, Yu-Jung Wu, Si-Tse Jiang, Ming-Zong Lai

**Affiliations:** 1 Institute of Molecular Biology, Academia Sinica, Taipei 11529, Taiwan, R.O.C; 2 Graduate Institute of Immunology, National Taiwan University, Taipei 10057, Taiwan, R.O.C; 3 National Laboratory Animal Center, National Applied Research Laboratories, Tainan 74147, Taiwan, R.O.C

## Abstract

Application of regulatory T cells (Tregs) in transplantation, autoimmunity and allergy has been extensively explored, but how Foxp3 and Treg stability is regulated *in vivo* is incompletely understood. Here, we identify a requirement for Deltex1 (DTX1), a contributor to T-cell anergy and Foxp3 protein level maintenance *in vivo*. *Dtx1*^*−/−*^ Tregs are as effective as WT Tregs in the inhibition of CD4^+^CD25^*−*^ T-cell activation *in vitro*. However, the suppressive ability of *Dtx1*^*−/−*^ Tregs is greatly impaired *in vivo*. We find that Foxp3 expression is diminished when *Dtx1*^*−/−*^ Tregs are co-transferred with effector T cells *in vivo*. DTX1 promotes the degradation of HIF-1α. Knockout of HIF-1α restores the Foxp3 stability and rescues the defective suppressive activity in *Dtx1*^*−/−*^ Treg cells *in vivo*. Our results suggest that DTX1 exerts another level of control on Treg stability *in vivo* by sustaining the expression of Foxp3 protein in Tregs.

## Abstract (web summary)

Foxp3, a key transcription factor of regulatory T cells, is targeted to degradation by HIF-1α. Here the authors show that Deltex1 promotes degradation of HIF-1α, thus stabilizing Foxp3 levels and function of regulatory T cells *in vivo*.

## Graphical abstract

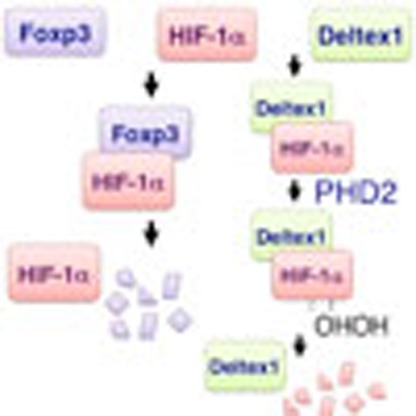

CD4^+^CD25^+^ regulatory T cells (Tregs) maintain T-cell tolerance and prevent autoimmune diseases by inhibiting T-cell activation^1^,^2^,^3^,^4^,^5^. The suppressive action of Tregs is known to proceed via many mechanisms, including cytolysis, secretion of suppressive cytokines, competition for cytokines, metabolic disruption of targeted cells and priming of dendritic cells^3^,^6^,^7^. Several Treg surface molecules, such as CTLA4, GITR, CD103, LAG3 and FR4, are also involved in the inhibition of target T-cell activation through cell–cell contact^8^,^9^,^10^. Tregs are classified into thymus-derived CD4^+^ CD25^+^Foxp3^+^ Tregs (tTregs), which develop in the thymus before their exit into the peripheral circulation, and induced Tregs (iTregs or converted Tregs), which may be generated by treatment with transforming growth factor (TGF)-β and interleukin (IL)-2 (refs 11, 12). Tregs are marked by the expression of Foxp3, a forkhead family transcription factor that is essential for their development and function^13^,^14^. In addition, the persistent presence of Foxp3 is required to maintain the effector activities of Tregs^15^,^16^. The expression and epigenetic control of *Foxp3* gene have been well characterized^17^. In contrast, regulation on the protein stability of Foxp3 remains poorly understood.

The potential application of Tregs in transplantation, autoimmune diseases and allergy are being extensively examined^18^,^19^,^20^,^21^. How Foxp3 and Treg stability are regulated *in vivo* remains incompletely understood. Different results are reported for the *in vivo* Treg stability^22^. Treg cells have been shown to be relatively stable *in vivo*, with a small population of Tregs susceptible to loss of Foxp3 (refs 23, 24). Alternatively, pathogen infection or inflammatory cytokines destabilize Foxp3 protein and decrease the Treg population^25^,^26^,^27^,^28^. Loss of Foxp3 in Tregs results in pathogenic autoimmunity manifested by the generation of inflammatory effector T cells^29^,^30^. Foxp3 protein is subjected to controlled degradation by several molecules. E3 ubiquitin ligase Stub1 induces the K48-polyubiquitination and degradation of Foxp3 (ref. 28), whereas deubiquitinase USP7 decreases Foxp3 polyubiquitination and increases *Foxp3* expression^31^. Hypoxia-inducible factor-1α (HIF-1α) binds Foxp3, inducing its degradation and thereby inhibiting Treg development^32^. Interfering with the binding of HIF-1α to Foxp3 increases Foxp3 protein stability and Treg suppressive activity^33^. Given the hypoxic conditions *in vivo*^34^, a mechanism should exist to prevent Foxp3 protein from HIF-1α-mediated destabilization *in vivo*.

Deltex (DTX) is a ubiquitin E3 ligase containing N-terminal Notch-binding WWE domains, a proline-rich motif and a RING finger (RF) domain at the C-terminus^35^,^36^. DTX mediates Notch activation in *Drosophila*^37^,^38^, but its exact role in Notch signalling in vertebrates remains unclear. We recently found that DTX1 negatively regulates T-cell activation by targeting MAP kinase kinase kinase 1 (MEKK1) for degradation^39^. DTX1 is a transcriptional target of nuclear factor of activated T cell (NFAT), is induced in T-cell anergy and inhibits T-cell activation in E3-dependent and E3-independent mechanisms^40^. DTX1-deficiency impairs T-cell anergy induction, promotes excess T-cell activation and generates autoimmunity^40^. In this study, we found that Treg-specific knockout of *Dtx1* also led to enhanced T-cell activation and autoantibody generation. Surprisingly, DTX1 deficiency did not affect the expression of *Foxp3*, nor was the suppressive activity of Treg affected *in vitro*. The *in vivo* suppressive function of Treg cells was largely impaired in the absence of DTX1, which was attributed to a diminished Foxp3 protein stability in *Dtx1*^*−/−*^ Tregs *in vivo*. We found that Foxp3 protein stability was enhanced by DTX1, and DTX1 induced degradation of HIF-1α and interfered with the capacity of HIF-1α to degrade Foxp3. Knockout of HIF-1α restored the Foxp3 expression and rescued the defective suppressive activity in *Dtx1*^*−/−*^ Treg cells *in vivo*. Our results illustrate a novel role for DTX1 in maintaining the protein stability of Foxp3 *in vivo* and reveal an additional level of control of Treg stability.

## Results

### Treg-specific deletion of Dtx1 enhances T-cell activation

**Figure 1:**
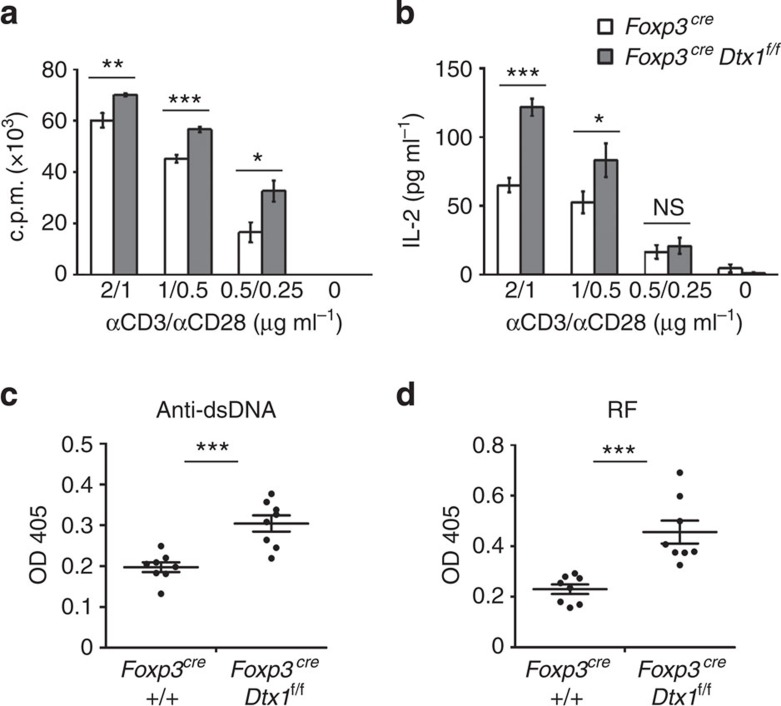
Conditional DTX1 deficiency in Tregs leads to enhanced T-cell activation and elevated autoantibody generation. (**a**,**b**) Treg-specific deletion of *Dtx1* resulted in enhanced T-cell activation. Lymph node T cells from control (*Foxp3*^*Cre*^) or *Foxp3*^*Cre*^ *Dtx1*^*f/f*^ mice were stimulated with plate-bound anti-CD3 plus anti-CD28. T-cell proliferation (**a**) was determined by [^3^H]thymidine incorporation 56 h after stimulation, and IL-2 production (**b**) was measured 40 h after activation. Error bars represent s.d. Data are mean±s.d. of triplicate samples from one mouse pair. Results were independently reproduced in five mouse pairs. (**c**,**d**) Elevated serum anti-DNA antibodies and rheumatoid factor in *Foxp3*^*Cre*^ *Dtx1*^*f/f*^ mice. Sera (1:100 dilution) from *Foxp3*^*Cre*^ *Dtx1*^*f/f*^ mice and *Foxp3*^*Cre*^ controls older than 6 months were analysed by anti-dsDNA (**c**) and anti-IgG1 (**d**) antibodies. *n*=8. *P<0.05; **P<0.01; ***P<0.001 (two-tailed unpaired *t*-test).

We previously demonstrated that T cell-specific deletion of *Dtx1* (*Dtx1*^*f/f*^*Cd4*^*Cre*^) leads to enhanced T-cell activation and autoantibody formation^40^. To examine the possible involvement of DTX1 in regulatory T-cell function, we generated mice with Treg-specific deletion of *Dtx1* by crossing *Dtx1*^*f/f*^ mice with *Foxp3*^*GFP-Cre*^ mice^41^ or *Foxp3*^*RFP-Cre*^ mice produced in this study. Treg cells were marked by green fluorescent protein (GFP) or red fluorescent protein (RFP) expression. No difference was found between *Foxp3*^*GFP-Cre*^ mice and *Foxp3*^*RFP-Cre*^ mice, and *Foxp3*^*Cre*^ was used to represent both. The selective deficiency of DTX1 in Foxp3^+^ T cells (tTregs), but not in Foxp3^*−*^ T cells from *Foxp3*^*Cre*^*Dtx1*^*f/f*^ mice, was confirmed by immunoblots (Supplementary Fig. 1a). Similar to mice with systemic and T cell-specific conditional knockout of *Dtx1* (ref. 40) thymic development was not disturbed by deficiency of DTX1 in Tregs (Supplementary Fig. 1b). Populations of splenic CD4^+^ and CD8^+^ T cells were comparable between control and *Foxp3*^*Cre*^*Dtx1*^*f/f*^ mice (Supplementary Fig. 1c). Neither was the naïve and memory T-cell ratio affected by Treg-specific absence of DTX1 (Supplementary Fig. 1d). However, T-cell proliferation and IL-2 production were elevated in T cells from *Foxp3*^*Cre*^*Dtx1*^*f/f*^ mice, relative to T cells from *Foxp3*^*Cre*^ mice (Fig. 1a,b). Small increases in interferon (IFN)-γ and IL-17 expression could be detected in naïve *Foxp3*^*Cre*^*Dtx1*^*f/f*^ T cells (Supplementary Fig. 1e). In addition, elevation of anti-dsDNA antibodies and rheumatoid factor (anti-IgG1) was also found in older *Foxp3*^*Cre*^*Dtx1*^*f/f*^ mice (>6-month old; Fig. 1c,d). Notably, the increase in T-cell activation in *Foxp3*^*Cre*^*Dtx1*^*f/f*^ T cells was less profound than in T cells from *Cd4*^*Cre*^*Dtx1*^*f/f*^ mice^40^. No increase in anti-histone antibodies was found in *Foxp3*^*Cre*^*Dtx1*^*f/f*^ mice (Supplementary Fig. 1f), in contrast to that seen in *Cd4*^*Cre*^*Dtx1*^*f/f*^ mice^40^. These results suggest that DTX1-deficiency in Treg accounts for part, but not all, of the phenotypes observed in *Cd4*^*Cre*^*Dtx1*^*f/f*^ mice, and DTX1 is required for the functional activities of Treg *in vivo*.

### Dtx1^*−*/*−*^ tTregs fail to suppress inflammatory bowel disease

**Figure 2:**
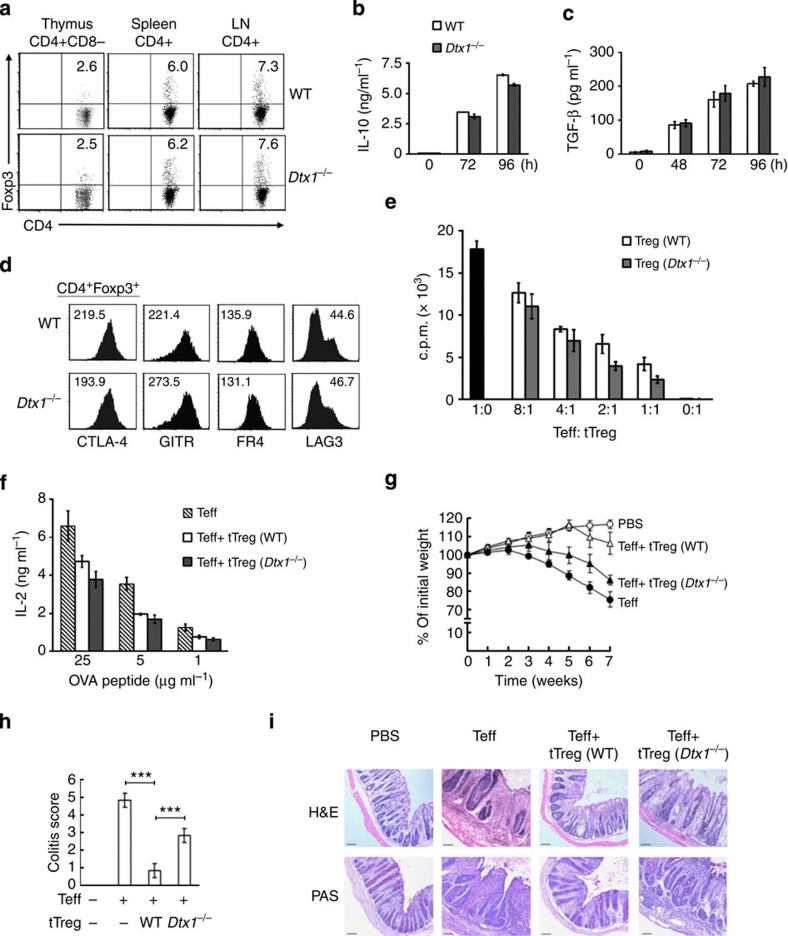
DTX1 deficiency impairs the suppressive activity of tTregs *in vivo.* (**a**) Normal CD4^+^Foxp3^+^ population in *Dtx1*^*−/−*^ mice. CD4^+^CD8^*−*^ or CD4^+^ population in thymocytes, splenocytes and lymph node cells were gated, and the fraction of Foxp3^+^ population determined by FACS. (**b**,**c**) Normal IL-10 and TGF-β secretion in *Dtx1*^*−/−*^ tTreg cells. CD4^+^CD25^+^ cells were stimulated with anti-CD3/CD28 plus IL-2, and the secretion of IL-10 (**b**) and TGF-β (**c**) were determined by ELISA. (**d**) DTX1 deficiency does not affect Treg phenotype. The expression of CTLA-4, GITR, FR4 and LAG3 in WT and *Dtx1*^*−/−*^ CD4^+^CD25^+^ cells were determined by flow cytometry. Number indicates mean fluorescence intensity. (**e**) Normal *in vitro* suppressive activity of *Dtx1*^*−/−*^ tTreg cells. Splenic CD4^+^CD25^*−*^ cells (1 × 10^5^) were incubated with anti-CD3 (1 μg ml^*−*1^), 3 × 10^5^ γ-irradiated T-depleted splenic cells and the indicated ratios of WT and *Dtx1*^*−/−*^ CD4^+^CD25^+^ tTreg cells. [^3^H]Thymidine incorporation was determined at 80 h. Values are mean±s.d. of triplicate samples in an experiment. (**f**) Normal antigen-specific suppression by *Dtx1*^*−/−*^ tTreg cells. tTreg cells (2.5 × 10^4^) from OT-II transgenic mice (WT) or *Dtx1*^*−/−*^ × OT-II mice were incubated with OT-II CD4^+^CD25^−^ cells (1 × 10^5^) in the presence of OVA and γ-irradiated T-depleted splenic cells. IL-2 production was measured 48 h later. Data shown are representative of five (**a**,**e**), three (**b**–**d**) or two (**f**) independent experiments. (**g**–**i**) Diminished inhibitory activity of *Dtx1*^*−/−*^ tTreg cells *in vivo*. CD4^+^CD25^−^ T cells (4 × 10^5^) or PBS were administered intraperitoneally into *Rag1*^*−/−*^ mice with or without 1 × 10^5^ tTregs. Body weight (**g**) was assessed weekly for 7 weeks. (**h**) Colitis was determined at the end of 7th week and scored as described in Methods. Data are the mean±s.d. of six mice in each group. ***P<0.001 (paired t-test). (**i**) Mice were killed and colons were removed, cleaned, fixed in paraformaldehyde, embedded in paraffin, sectioned and stained with haematoxylin and eosin (H&E) and periodic acid–Schiff (PAS). Micrographs are representative of the six mice in each group. Scale bar, 100 μm.

The observations that Treg-conditional knockout of DTX1 led to enhanced T-cell activation and autoantibody production were unexpected, because we have previously found that DTX1 deficiency did not affect the development of tTreg^40^, and the frequency of CD4^+^Foxp3^+^ cells in thymus, spleen and lymph node was comparable between *Dtx1*^*−/−*^ mice and littermate control (wild-type (WT)) animals (Fig. 2a). The production of IL-10 and TGF-β in *Dtx1*^*−/−*^ tTreg cells was indistinguishable from that in WT tTreg (Fig. 2b,c). We also determined the expression of several Treg-associated molecules in isolated WT and *Dtx1*^*−/−*^ tTreg cells; CTLA-4, GITR, FR4, LAG3, CD103, ICOS and surface TGF-β expression were all comparable between WT and *Dtx1*^*−/−*^ tTreg cells (Fig. 2d and Supplementary Fig. 2a,b). Furthermore, in an *in vitro* suppression analysis, *Dtx1*^*−/−*^ tTreg cells were as effective as Tregs from WT littermate controls in suppressing the proliferation of T-cell antigen receptor (TCR)-stimulated CD4^+^CD25^*−*^ T cells in proportion to the number of tTreg cells added (Fig. 2e). Recent studies have also revealed differences between polyclonal and monoclonal Treg populations in the suppression mechanism^5^. We examined antigen-specific Treg suppression, and found no difference between OT-2 Tregs and OT-2*/Dtx1*^*−/−*^ Tregs in the inhibition of OT-2 effector T cells (Fig. 2f).

However, in an *in vivo* functional assay, *Dtx1*^*−/−*^ tTregs displayed a diminished capacity to suppress T-cell activation. Colitis was induced in *Rag1*^*−/−*^ mice by administration of CD4^+^CD25^*−*^ T cells, leading to 20–30% weight loss, rectal prolapse and diarrhoea (Fig. 2g,h). Tissue sections of inflamed colon revealed inflammatory infiltrate, epithelial hyperplasia, crypt cell damage and goblet cell damage (Fig. 2i). Co-injection with WT tTregs effectively repressed the induction of colitis by CD4^+^CD25^*−*^ T cells. In contrast, the co-administration of *Dtx1*^*−/−*^ tTregs only weakly reduced the colitis-associated pathogenesis (Fig. 2g–i), suggesting that the absence of DTX1 greatly diminished the suppressive activities of tTregs *in vivo*. Therefore, even though the expression of DTX1 is dissociated from the development and *in vitro* activities of tTregs, DTX1 is indispensable for the *in vivo* function of tTregs.

### Dtx1^*−*/*−*^ iTreg cells fail to inhibit allergic inflammation

**Figure 3:**
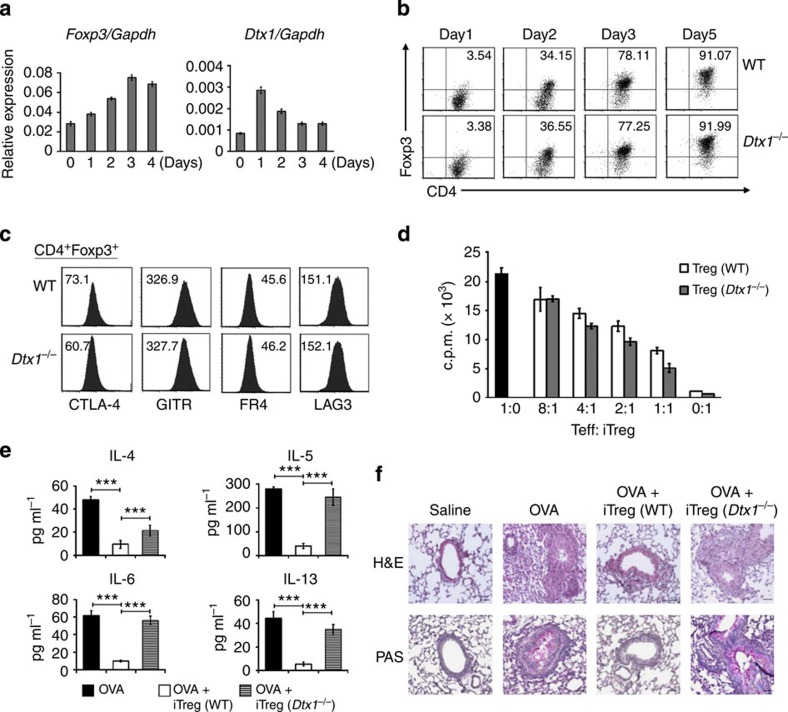
*Dtx1*^*−/−*^ iTreg cells are functionally defective *in vivo*. (**a**) Induction of *Dtx1* and *Foxp3* during iTreg differentiation. CD4^+^CD25^−^ T cells from WT mice were treated with immobilized anti-CD3/CD28 in the presence of TGF-β (5 ng ml^−1^) and IL-2 (20 ng ml^−1^) for 4 days, and the expression of the transcripts of *Dtx1* and *Foxp3* determined by quantitative PCR. (**b**,**c**) Differentiation of iTregs and expression of Foxp3 were not affected by DTX1 deficiency. CD4^+^CD25^−^ T cells from WT and *Dtx1*^*−/−*^ mice were treated as in **a**, and Foxp3 expression at days 1, 2, 3 and 5 was assessed by intracellular staining (**b**). The expression of CTLA-4, GITR, FR4 and LAG3 of iTregs at day 5 was determined (**c**). Numbers indicate mean fluorescence intensity. (**d**) Normal *in vitro* suppressive activity of *Dtx1*^*−/−*^ iTreg cells. CD4^+^CD25^−^ T cells from WT and *Dtx1*^*−/−*^ T cells were differentiated into iTregs for 5 days. Splenic CD4^+^CD25^−^ cells (1 × 10^5^) were activated in the presence of the indicated ratio of CD4^+^CD25^+^ iTreg cells described in Fig. 2e. [^3^H]thymidine incorporation was determined at 80 h. Results are the mean±s.d. of triplicate samples from a single experiment. Experiments were reproduced independently three times for **b**–**d** and twice for **a**. (**e**,**f**) Diminished *in vivo* inhibitory activity of *Dtx1*^*−/−*^ iTreg cells. C57/BL6 mice were treated with CD4^+^CD25^+^ iTreg cells or PBS on day 0, and primed with 50 μg ovalbumin (OVA) in alum on days 1 and 14. Mice were challenged with aerosolized 1% OVA by airway from days 21 to 24. Mice were killed on day 25 and bronchoalveolar lavage was collected. The contents of IL-4, IL-5, IL-6 and IL-13 were determined by Cytometric Bead Array kit (**e**). Sections of lung were examined after staining with H&E and PAS (**f**). ***P<0.001 (paired *t*-test). Micrographs are representative of the six mice in each group. Scale bar, 50 μm.

In addition to tTregs, Tregs could be generated from CD4^+^CD25^*−*^ T cells by incubation with anti-CD3/anti-CD28 and TGF-β. Both DTX1 and Foxp3 were induced in CD4^+^CD25^*−*^ T cells treated with anti-CD3/anti-CD28 in the presence of TGF-β, with an earlier peak in upregulation of DTX1 than Foxp3 during iTreg differentiation (Fig. 3a). Despite that DTX1 was induced before Foxp3 in iTregs, the absence of DTX1 did not affect generation of iTregs from CD4^+^CD25^*−*^ T cells. During the differentiation of iTregs, the induction of Foxp3 was nearly identical between WT and *Dtx1*^*−/−*^ T cells (Fig. 3b). Together with the fact that Foxp3 levels were comparable between WT and *Dtx1*^*−/−*^ tTregs (Fig. 2), it is clear that DTX1 does not regulate the expression of Foxp3 during the development of Tregs in the thymus or periphery. In addition, no differences in the expression of CTLA-4, GITR, FR4 or LAG3 could be found between WT and *Dtx1*^*−/−*^ iTreg cells (Fig. 3c). We also analysed the *in vitro* suppressive activity of iTregs. Both WT iTregs and DTX1-KO iTregs inhibited the activation of CD4^+^CD25^*−*^ T cells to the same extent (Fig. 3d). Similar to tTregs, DTX1 deficiency did not affect the suppressive function of iTregs *in vitro*.

We further examined the *in vivo* regulatory function of iTregs. Lung inflammation was examined by bronchoalveolar lavage (BAL). Allergen-induced IL-4 secretion in BAL was suppressed by WT iTregs but was less effectively inhibited by *Dtx1*^*−/−*^ iTregs (Fig. 3e). WT iTregs inhibited ovalbumin (OVA)-mediated secretion of IL-5, IL-6 and IL-13 in BAL, whereas *Dtx1*^*−/−*^ iTregs failed to prevent the production of these cytokines. OVA sensitization also led to massive inflammatory cell infiltration and mucus obstruction of the airway upon OVA re-challenge (Fig. 3f). Pre-administration of WT iTregs prevented this exacerbation of the allergic response (Fig. 3f, OVA+WT iTreg). In contrast, *Dtx1*^*−/−*^ iTregs cells were unable to suppress the profound immune reaction triggered by allergen re-challenge (Fig. 3f, OVA+*Dtx1*^*−/−*^ iTreg). These results illustrate that DTX1 is also required for the *in vivo* suppressive function of iTregs.

### Downregulation of Foxp3 in transferred Dtx1^*−*/*−*^ tTreg cells

The prominent role of DTX1 in the *in vivo* Treg effector function prompted further search for possible defects in *Dtx1*^*−/−*^ Treg cells. The expression of *Foxo1*, *Foxo3a*, *Ikzf4* (encoding Eos), *Ebi3* (encoding IL-35 subunit) and *Gzmb* (encoding granzyme B) was similar in tTreg cells from WT and DTX1-null mice (Supplementary Fig. 2c–e). We also examined whether the expression of chemokine receptors, required for T-cell homing *in vivo*, was altered by DTX1 deficiency. The transcripts of *Ccr4*, *Ccr6* and *Ccr7*, as well as the surface amounts of CCR4, CCR5, and CCR6, were nearly identical between WT and *Dtx1*^*−/−*^ tTreg cells isolated from spleen and mesentery lymph nodes (Supplementary Fig. 3). DTX1 deficiency thus did not affect the expression of several chemokine receptors on Treg cells. We also examined activation-induced cell death in WT and *Dtx1*^*−/−*^ tTreg. No difference was found in the extent of cell death (Annexin-V^+^ and PI^+^) induced by CD3 stimulation between these two types of tTregs (Supplementary Fig. 2f). Because the *in vivo* conditions for iTreg differentiation is not necessarily optimal, we additionally determined whether the conversion of CD4^+^ naïve T cells into iTreg cells at low TGF-β concentration was specifically affected by DTX1 deficiency. Only a very small decease in Foxp3 levels was found in the generation of *Dtx1*^*−/−*^ iTreg cells at suboptimal TGF-β concentrations, relative to WT iTreg cells (Supplementary Fig. 4a).

**Figure 4:**
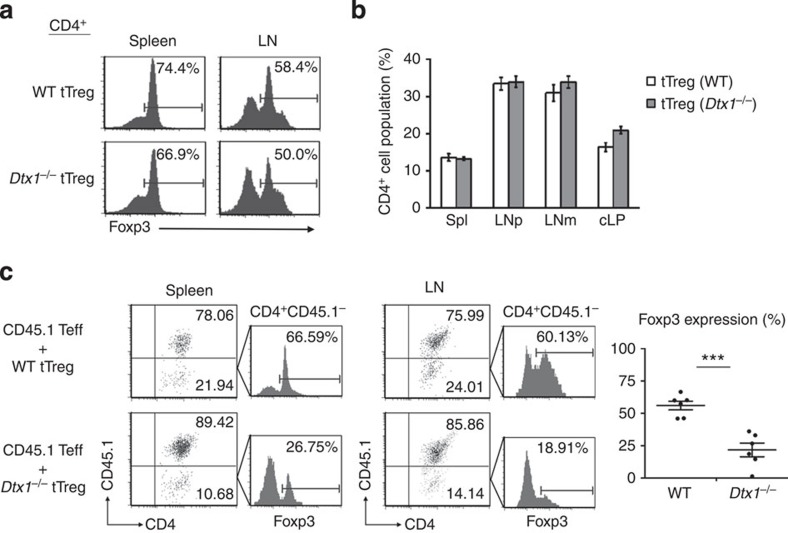
Co-transfer of *Dtx1*^*−/−*^ tTregs with effector T cells leads to Foxp3 downregulation. (**a**) DTX1 deficiency did not affect the Foxp3^+^ population when tTregs were transferred alone. We administered 1 × 10^5^ tTreg cells from WT and *Dtx1*^*−/−*^ mice into *Rag1*^−/−^ mice. Spleen and lymph nodes were isolated 1 week later, and the fraction of Foxp3^+^ cells in CD4^+^ T cells was determined. (**b**) A comparable CD4^+^ population in mice that received WT or *Dtx1*^*−/−*^ tTregs. *Rag1*^*−/−*^ mice were adoptively transferred with WT or *Dtx1*^*−/−*^ CD4^+^CD25^+^ tTreg cells. Spleen, peripheral lymph nodes (LNp), mesenteric lymph nodes (LNm) and colonic lamina propria (cLP) were isolated from the recipient mice a week later. Frequencies of CD4^+^ tTreg cells were analysed by flow cytometry. (**c**) Diminished Foxp3^+^ population from *Dtx1*^*−/−*^ tTregs adoptively transferred with CD4^+^CD25^−^ cells. CD4^+^CD25^−^ T cells from CD45.1^+^ mice were co-transferred with 1 × 10^5^ WT or *Dtx1*^*−/−*^ tTreg cells (CD45.2^+^) into *Rag1*^−/−^ mice. Spleen and lymph nodes were isolated 1 week later, and the fraction of Foxp3^+^ cells in the CD4^+^CD45.1^−^ T-cell population was determined. The percentage of Foxp3^+^ cells from spleen and lymph nodes in WT and *Dtx1*^*−/−*^ CD4^+^CD45.1^−^ T cells is compared from three pair of mice (right panel). ***P<0.001 (paired t-test).

Because iTregs may not be able to maintain stable Foxp3 expression *in vivo*^42^,^43^, tTregs were used for further investigation of *in vivo* stability. To examine the consequences of defective *Dtx1*^*−/−*^ tTregs *in vivo*, WT or *Dtx1*^*−/−*^ tTreg cells were transferred into *Rag1*^*−*/*−*^ mice, and the fraction of CD4^+^ T cells with Foxp3 expression in spleen and lymph nodes was determined 1 week after transfer. The recovered CD4^+^Foxp3^high^ population was similar for WT and *Dtx1*^*−/−*^ tTregs (Fig. 4a). The CD4^+^ T-cell frequency repopulating different lymph tissues of *Rag1*^*−*/*−*^ mice was comparable after the transfer of WT and *Dtx1*^*−/−*^ tTregs (Fig. 4b). We then examined the effect of CD4^+^CD25^*−*^ T-cell co-transfer on the *in vivo* stability of tTregs. CD45.2^+^ tTregs were co-administered with CD45.1^+^ effector T cells into *Rag1*^*−*/*−*^ mice. One week later, the CD45.2^+^Foxp3^high^ populations in spleen and lymph nodes of the recipient mice were quantitated. A large percentage of WT tTreg cells retained high levels of Foxp3 expression when co-transferred with CD4^+^CD25^*−*^ T cells (Fig. 4c). In contrast, the Foxp3^high^ population from mice receiving *Dtx1*^*−/−*^ tTregs was profoundly reduced (Fig. 4c). In contrast to the stability of WT Tregs *in vivo*, DTX1 is required for the *in vivo* stability of Tregs when T effector cells were present. In addition, small increases in the expression of IFN-γ, IL-17, TNF-α and IL-4 in the *Dtx1*^*−/−*^ tTreg cells recovered from *Rag1*^*−*/*−*^ mice were found (Supplementary Fig. 4b), suggesting that some portions of the tTreg population may be converted into effector cells *in vivo*.

### DTX1 promotes HIF-1α degradation

Because the *in vivo* stability of Foxp3 was compromised in the presence of co-transferred CD4^+^CD25^*−*^ T cells, we speculate that inflammation associated with effector T cells contributes to Foxp3 downregulation. We examined whether *Dtx1*^*−/−*^ Tregs behaved differently from WT Tregs in the inflammatory mucosal environments where colitis and allergic airway responses take place. Retinoic acid is known to promote the differentiation of Tregs *in vivo* at mucosal sites that are often hypoxic^44^,^45^. WT and *Dtx1*^*−/−*^ CD4^+^ T cells responded similar to retinoic acid in TGF-β-induced iTreg differentiation (Supplementary Fig. 5a). *Dtx1*^*−/−*^ iTregs generated in the presence of retinoic acid produced IL-10 and TGF-β indistinguishably from WT iTregs (Supplementary Fig. 5b) and suppressed the activation of effector T cells similar to retinoic acid-treated WT iTregs, as measured by the secretion of IFN-γ and IL-2 by effector T cells (Supplementary Fig. 5c). DTX1 deficiency thus did not affect the ability of retinoic acid to stabilize the Treg population.

Recent studies demonstrate that Foxp3 protein could be destabilized by incubation with inflammatory cytokines *in vitro*^25^,^26^,^27^,^28^. We examined whether DTX1 regulated the stability of Foxp3 in Treg cells encountering inflammatory cytokines. WT and *Dtx1*^*−/−*^ tTreg cells were treated with inflammatory cytokines and lipopolysaccharide (LPS). Similar to those reported, tTregs displayed variable sensitivity to different inflammatory cytokines *in vitro*, shown by reduction in the Foxp3 protein levels (Supplementary Fig. 6a). No difference in inflammatory cytokine-induced Foxp3 downregulation was observed between WT and *Dtx1*^*−/−*^ tTregs (Supplementary Fig. 6a).

**Figure 5:**
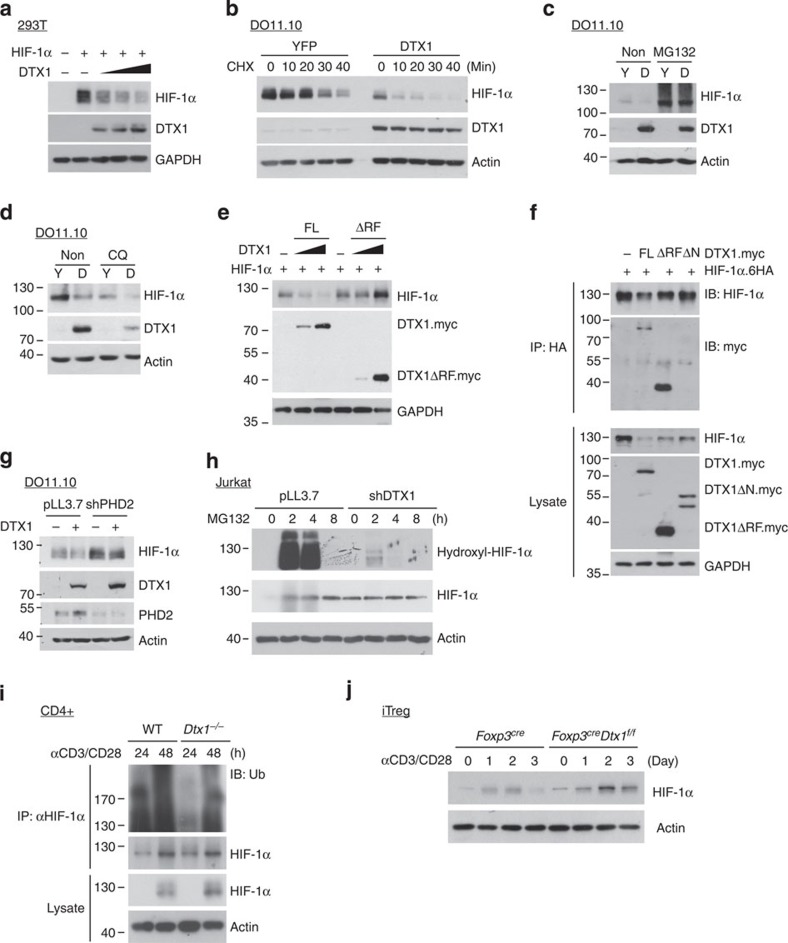
DTX1 promotes HIF-1α downregulation. (**a**) DTX1 promotes HIF-1α downregulation. 293T cells were transfected with HIF-1α and DTX1 (0.5, 1 and 2 μg), and cellular levels of HIF-1α were determined 48 h later. (**b**) DTX1 decreases the stability of HIF-1α protein in T cells. Control and DTX1-expressing DO11.10 T cells were incubated in hypoxic conditions (1.1% O_2_) for 24 h. Cells were then treated with cycloheximide (CHX), and the amounts of HIF-1α and DTX1 monitored. (**c**,**d**) DTX1-induced HIF-1α degradation in T cells is proteasome dependent. YFP control and DTX1-expressing DO11.10 cells were incubated in hypoxic conditions for 4 h, in the presence or absence of 10 μM MG132 (**c**) or 50 nM chloroquine (**d**), and the levels of HIF-1α and DTX1 analysed. (**e**) The RING finger domain is required for DTX1 to promote HIF-1α downregulation. HIF-1α was co-expressed with DTX1 (FL) or DTX1ΔRF in 293T cells, and the levels of HIF-1α, DTX1 or DTX1ΔRF were analysed. (**f**) Interaction between DTX1 and HIF-1α. 293T cells were transfected with DTX1(FL), DTX1ΔRF or DTX1ΔN, and HIF-1α-6HA. The cell lysates were immunoprecipitated using anti-HA, and were analysed with anti-Myc and anti-HIF-1α. ΔN, N-terminal WWE domain deletion. (**g**) PHD2 knockdown attenuates DTX1-mediated HIF-1α downregulation. Control (pLL3.7) and PHD2-knocked down DO11.10 cells with or without DTX1 overexpression were incubated in hypoxic conditions for 4 h, and the contents of HIF-1α, DTX1 and PHD2 were determined. (**h**) DTX1-KD increases HIF-1α proline hydroxylation. Jurkat cells were treated with MG132 and the extent of proline hydroxylation on HIF-1α was determined. (**i**) Decreased HIF-1α ubiquitination in *Dtx1*^*−/−*^ T cells stimulated by CD3/CD28. WT and *Cd4*^*Cre*^ *Dtx1*^*f/f*^ CD4^+^ T cells were stimulated by CD3/CD28, and total cell lysates isolated at 24 and 48 h after activation. Cell lysates were precipitated with anti-HIF-1α, and the extent of ubiquitination was determined by anti-Ub. (**j**) Increased HIF-1α expression in *Dtx1*^*−/−*^ iTreg cells stimulated by CD3/CD28. *Foxp3*^*Cre*^ and *Foxp3*^*Cre*^*Dtx1*^*f/f*^ iTreg cells were generated as described in Fig. 3b. iTreg cells were subjected to activation by CD3/CD28 in normoxic conditions, and HIF-1α expression was monitored. All experiments were independently repeated three (**a**–**g**) or two (**h**–**j**) times. GAPDH, glyceraldehyde 3-phosphate dehydrogenase; HA, hemagglutinin tag; IB, immunoblotting; IP, immunoprecipitation.

In the hypoxic or inflammatory tissues, HIF-1α is one of the few proteins suggested to destabilize Foxp3 by binding Foxp3 and stimulating the ubiquitination of Foxp3, leading to reduction in Treg population^32^. We identified an antagonism between DTX1 and HIF-1α, observing that expression of increasing amounts of DTX1 resulted in decreased HIF-1α protein levels (Fig. 5a). The exposure of DO11.10 T cells to hypoxic conditions upregulated HIF-1α levels, whereas co-expression of DTX1 greatly decreased the protein half-life of HIF-1α (Fig. 5b). DTX1-mediated downregulation of HIF-1α was prevented by treatment with proteasome inhibitor MG132 (Fig. 5c), but not by chloroquine (Fig. 5d) in DO11.10 T cells. Deletion of the RF domain of DTX1 attenuated the ability of DTX1 to promote HIF-1α destabilization (Fig. 5e), indicating a critical role of the RF domain of DTX1 in the downregulation of HIF-1α.

We further detected an interaction of DTX1 with HIF-1α. Immunoprecipitation of HIF-1α brought down DTX1 in 293T cells (Fig. 5f). The interaction between DTX1 and HIF-1α was independent of the RF of DTX1, as shown by the association of HIF-1α with DTX1ΔRF (Fig. 5f). The N-terminal WWE domain, in contrast, was required for the interaction between HIF-1α and DTX1 (Fig. 5f). No interaction between DTX1 and Foxp3, however, was detected (Supplementary Fig. 6b). Despite the binding of DTX1 to HIF-1α, DTX1 did not directly induce the ubiquitination of HIF-1α, as illustrated by *in vitro* ubiquitination analysis (Supplementary Fig. 7). The stability of HIF-1α is regulated by prolyl hydroxylase domain protein (PHD)-mediated hydroxylation at Pro402 and Pro564, located in the O_2_-dependent degradation domain (ODD) region^46^. Knockdown of PHD2 prevented DTX1-induced HIF-1α degradation in DO11.10 T cells (Fig. 5g), suggesting that DTX1-induced HIF-1α downregulation is mediated by a PHD2-dependent pathway. MG132 restored HIF-1α in normoxic T cells (Fig. 5c), whereas DTX1 deficiency decreased the proline hydroxylation of HIF-1α in MG132-treated Jurkat T cells (Fig. 5h). Prolyl hydroxylated HIF-1α is recognized by the von Hippel–Lindau (VHL)-containing E3 complex and ubiquitinated for proteasome degradation^46^. By using the induction of HIF-1α in normal T cells by TCR/CD28 ligation under normoxic conditions, we found that DTX1 deficiency decreased HIF-1α ubiquitination in CD4^+^ T cells activated through CD3/CD28 (Fig. 5i). We also observed that CD3/CD28-induced HIF-1α expression was increased in *Dtx1*^*−/−*^ iTreg cells relative to WT iTreg cells (Fig. 5j). DTX1 did not affect the protein stability of PHD2 and VHL (Supplementary Fig. 8). We further found an association between DTX1 and PHD2 (Supplementary Fig. 9a). DTX1, but not DTX1ΔRF, inhibited the oligomerization of PHD2 (Supplementary Fig. 9b). How the suppression of PHD2 oligomerization by DTX1 leads to HIF-1α degradation is being delineated. Together, these results suggest that DTX1 promotes HIF-1α degradation by binding to HIF-1α and increasing PHD2-mediated proline hydroxylation and the subsequent ubiquitination of HIF-1α in T cells.

### DTX1 increases Foxp3 levels by antagonizing HIF-1α

**Figure 6:**
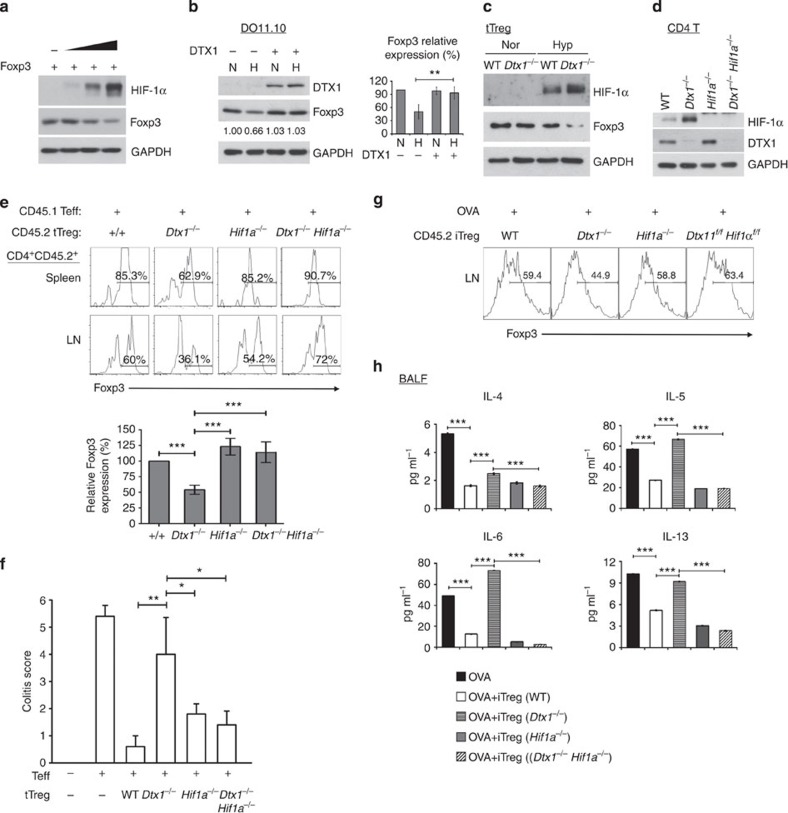
DTX1 increases Foxp3 stability through HIF-1α downregulation. (**a**) HIF-1α promotes Foxp3 degradation. 293T cells were transfected with Foxp3, ubiquitin and HIF-1α, and cellular levels of Foxp3 determined. (**b**) DTX1 protects Foxp3 from HIF-1α-induced degradation. Foxp3-expressing DO11.10 cells were transfected with or without DTX1. Cells were incubated in normoxic (N) conditions for 16 h and kept at N conditions or switched to hypoxic (H) conditions for 4 h, and levels of DTX1 and Foxp3 were analysed. Right panel, quantification from three independent experiments. (**c**) Increased HIF-1α induction and decreased Foxp3 levels in hypoxia-treated *Dtx1*^*−/−*^ tTreg cells. Control and *Dtx1*^*−/−*^ tTregs were activated by CD3/CD28 plus IL-2 in N or H conditions for 72 h, and the levels of HIF-1α and Foxp3 were determined. (**d**) Expression of DTX1 and HIF-1α in CD4^+^ T cells from WT, *Dtx1*^*−/−*^, *Hif1a*^*−/−*^ and *Dtx1*^*−/−*^*Hif1a*^*−/−*^ mice. (**e**) Reduced Foxp3^+^ population in transferred *Dtx1*^*−/−*^ tTregs are restored by HIF-1α deficiency. CD45.2^+^ WT (+/+), *Dtx1*^*−/−*^, *Hif1a*^*−/−*^ or *Dtx1*^*−/−*^*Hif1a*^*−/−*^ tTreg cells were co-transferred with CD45.1^+^ CD4^+^CD25^−^ T cells into *Rag1*^−/−^ mice. Spleen and lymph nodes were isolated 1 week later, and the frequency of Foxp3^+^ cells in the CD4^+^CD45.2^+^ T-cell population was determined. Bottom panel, splenic Foxp3^+^ cells from three mice each group. The percentage of +/+ was set as 100%. (**f**) *Hif1a* knockout enables *Dtx1*^*−/−*^ tTregs to inhibit colitis. Colitis was scored in *Rag1*^−/−^ mice transferred with effector T cells, and WT (*Foxp3*^*Cre*^), *Foxp3*^*Cre*^*Dtx1*^*f/f*^, *Foxp3*^*Cre*^*Hif1a*^*f/f*^ or *Foxp3*^*Cre*^*Dtx1*^*f/f*^*Hif1a*^*f/f*^ tTreg cells. (**g**) Decreased Foxp3^+^ population in transferred *Dtx1*^*−/−*^ iTregs are reversed by HIF-1α deficiency. CD45.2 WT (*Foxp3*^*Cre*^), *Foxp3*^*Cre*^*Dtx1*^*f/f*^, *Foxp3*^*Cre*^*Hif1a*^*f/f*^ or *Foxp3*^*Cre*^*Dtx1*^*f/f*^*Hif1a*^*f/f*^ iTreg cells were administered to CD45.1 B6 mice, followed by ovalbumin immunization. The transferred CD45.2 iTreg cells were isolated from lymph nodes of mice killed at day 25, and the frequency of Foxp3^+^ cells was determined. Data are representative of three mice in each group. (**h**) HIF-1α deficiency restores the *in vivo* inhibitory activity of *Dtx1*^*−/−*^ iTreg cells. B6 mice were treated with WT, *Dtx1*^*−/−*^ (*Cd4*^*Cre*^*Dtx1*^*f/f*^), *Hif1a*^*−/−*^ (*Cd4*^*Cre*^*Hif1a*^*f/f*^), *Dtx1*^*−/−*^*Hif1a*^*−/−*^ (*Cd4*^*Cre*^*Dtx1*^*f/f*^*Hif1a*^*f/f*^) iTreg cells or PBS as in Fig. 3. The contents of cytokines in the BAL were determined. GAPDH, glyceraldehyde 3-phosphate dehydrogenase. BALF, bronchoalveolar lavage fluid. *P<0.05, **P<0.01, ***P<0.001 for paired t-test.

We confirmed previous reports showing overexpression of HIF-1α resulted in a decrease in the levels of Foxp3 (Fig. 6a). In contrast, DTX1 expression led to an elevation of Foxp3 protein contents in 293T cells (Supplementary Fig. 6c). Hypoxia-induced HIF-1α upregulation was accompanied by a reduction in Foxp3 protein, whereas the expression of DTX1 prevented hypoxia-induced Foxp3 downregulation in DO11.10 T cells (Fig. 6b). We also examined the effect of hypoxia treatment on Foxp3 protein levels in tTregs. Foxp3 contents were comparable between control and *Dtx1*^*−/−*^ tTregs incubated with IL-2 under normoxic conditions (Fig. 6c). HIF-1α was induced in tTreg cells when switched to hypoxic conditions. DTX1 deficiency increased hypoxia-mediated HIF-1α upregulation in tTregs, with a concomitant decrease in Foxp3 (Fig. 6c). Together, these results suggest a protective effect of DTX1 against HIF-1α-mediated Foxp3 instability.

If the failure of *Dtx1*^*−/−*^ Tregs to inhibit effector T cells *in vivo* (Figs 2 and 3) were due to HIF-1α-mediated Foxp3 downregulation, we hypothesized that the additional deletion of HIF-1α should rescue the *in vivo* expression of Foxp3 and the function of *Dtx1*^*−/−*^ Tregs. We bred *Cd4*^*Cre*^*Hif1a*^*f/f*^ mice with *Cd4*^*Cre*^*Dtx1*^*f/f*^ mice to produce mice with *Dtx1*^*−/−*^*Hif1a*^*−/−*^ T cells and crossed *Foxp3*^*Cre*^*Hif1a*^*f/f*^ mice with *Foxp3*^*Cre*^*Dtx1*^*f/f*^ mice to generate *Dtx1*^*−/−*^*Hif1a*^*−/−*^ Treg cells (Fig. 6d). CD4^+^CD25^+^ T cells were isolated from *Cd4*^*Cre*^ mice (Supplementary Fig. 10), whereas RFP^+^ (tTreg) cells were sorted from *Foxp3*^*Cre*^ mice, and these tTreg cells were co-transferred with CD45.1^+^ effector T cells into *Rag1*^*−*/*−*^ mice. Foxp3 levels were not decreased in WT and *Hif1a*^*−/−*^ tTreg cells 1 week after transfer, in contrast to reduced Foxp3 expression in *Dtx1*^*−/−*^ tTregs at the same time point (Fig. 6e and Supplementary Fig. 11). The deletion of *Hif1a* restored the expression of Foxp3 in *Dtx1*^*−/−*^ tTregs *in vivo*, suggesting that HIF-1α accounts for the Foxp3 downregulation in DTX1-deficient Tregs. Consistent with normal levels of Foxp3 in the recovered Tregs, *Hif1a-*knockout re-established the ability of *Dtx1*^*−/−*^ tTregs to repress CD4^+^CD25^*−*^ T cell-induced colitis in *Rag1*^*−*/*−*^ mice as determined by colitis score readout (Fig. 6f), body weight loss and intestinal morphology (Supplementary Fig. 12). Histological analysis revealed that *Dtx1*^*−/−*^*Hif1a*^*−/−*^ tTregs efficiently inhibited CD4^+^CD25^*−*^ T cell-induced mononuclear cell infiltrate, crypt damage and goblet cell damage in *Rag1*^*−*/*−*^ mice, in contrast to the ineffectiveness of *Dtx1*^*−/−*^ tTregs (Supplementary Fig. 13).

We further examined the effect of HIF-1α-knockout in the suppressive activity of *Dtx1*^*−/−*^ iTregs. Reduction in Foxp3 expression was observed in *Dtx1*^*−/−*^ iTreg cells isolated from OVA-sensitized mice, and was reversed by additional *Hif1a-*knockout (Fig. 6g). *Hif1a*^*−/−*^ iTregs were as effective as WT iTregs in the inhibition of allergen-induced production of IL-5, IL-6 and IL-13 (Fig. 6h). The attenuated ability of *Dtx1*^*−/−*^ iTregs to suppress the allergenic IL-5, IL-6 and IL-13 secretion was rescued by *Hif1a* knockout, shown by the full inhibitory activity of *Dtx1*^*−/−*^*Hif1a*^*−/−*^ iTregs in the same assay. Lung tissue sections revealed that the extensive OVA-induced mononuclear cell infiltration was effectively prevented by administration of *Dtx1*^*−/−*^*Hif1a*^*−/−*^ iTregs (Supplementary Fig. 14). Together, these results indicate that deficiency in HIF-1α corrected the defects of *Dtx1*^*−/−*^ iTregs in inhibition of allergic inflammation. Therefore, part of the DTX1-mediated protective effects on Foxp3 expression *in vivo* is through antagonizing HIF-1α.

## Discussion

T cell-specific deletion of *Dtx1* results in increased T-cell activation and autoantibody generation^40^. In the present study, we further found that Treg-specific deletion of *Dtx1* led to enhanced T-cell activation and elevated autoantibody production (Fig. 1), suggesting that the presence of DTX1 in Tregs is also required for maintenance of full T-cell immune tolerance. The phenotypes of *Foxp3*^*Cre*^*Dtx1*^*f/f*^ mice were not as profound as those in *Cd4*^*Cre*^*Dtx1*^*f/f*^ mice. The increase in T-cell proliferation was modest in *Foxp3*^*Cre*^*Dtx1*^*f/f*^ T cells (Fig. 1a) relative to a twofold enhanced T-cell proliferation in *Cd4*^*Cre*^*Dtx1*^*f/f*^ T cells^40^. In addition, the elevated anti-histone antibodies in *Cd4*^*Cre*^*Dtx1*^*f/f*^ mice were not detectable in *Foxp3*^*Cre*^*Dtx1*^*f/f*^ mice (Supplementary Fig. 1f). Therefore, the phenotypes previously observed in *Cd4*^*Cre*^*Dtx1*^*f/f*^ mice^40^ are likely a combinatory consequence of the loss of T-cell anergy and impairment of Treg activity.

The normal development of tTregs in *Dtx1*^*−/−*^ mice and the comparable Foxp3 levels in *Dtx1*^*−/−*^ iTregs indicate that DTX1 does not directly participate in the expression of Foxp3. In addition, DTX1 deficiency did not affect the expression of Treg-associated molecules including CD25, CTLA-4 and GITR, which are also transcriptional targets of Foxp3. Unlike transcription factors like NFAT that are associated with Foxp3 for the expression of targets such as *Il2ra* and *Ctla4* (ref. 47), DTX1 is apparently not involved in the expression of genes through coordination with Foxp3.

We also demonstrated that Treg-specific deficiency in DTX1 impaired the suppressive function of tTregs and iTregs *in vivo*, but did not affect the ability of Tregs to inhibit T effector cell activation *in vitro*. Recent studies have shown that deficiency in STAT3 or Ubc13 does not affect the frequency of tTregs, the expression level of Foxp3 or the *in vitro* suppressive activity of Tregs, but profoundly alters the *in vivo* suppressive activity of tTregs^27^,^48^. For Treg function, various mechanisms have been identified for the Treg-mediated inhibitory mechanism *in vitro*. However, whether these mechanisms also operate *in vivo* remains unclear^7^. Our results thus provide additional evidence that the *in vivo* function of Tregs could be regulated by mechanisms not detectable *in vitro*.

We further found that the defective *in vivo* suppressive activity by *Dtx1*^*−/−*^ Treg cells could be attributed to the instability of Foxp3 protein. The expression and epigenetic control of the Foxp3 gene have been well characterized^17^. In contrast, the regulation on the Foxp3 protein stability is incompletely understood. Loss of Foxp3 in Tregs leads to generation of autoreactive effector T cells^29^,^30^. Two ubiquitin-modifying enzymes, Stub1 and USP7, act directly on Foxp3 protein stability. LPS and proinflammatory cytokines induce expression of E3 ubiquitin ligase Stub1, whereas Stub1 binds Foxp3 and induces Foxp3 K48-polyubiquitination and degradation in an Hsp70-dependent manner^28^. In contrast, the binding of deubiquitinase USP7 to Foxp3 promotes the removal of polyubiquitination from Foxp3 and increases Foxp3 protein levels^31^. In addition, non-ubiquitin-modifying enzymes like HIF-1α bind Foxp3 and promote Foxp3 degradation in PHD 2-dependent processes^32^, whereas Sirtuin 1 interacts with Foxp3 and promotes Foxp3 ubiquitination and degradation by deacetylation of Foxp3 (ref. 49).

In the present study, we demonstrated a new mechanism: the E3 ligase DTX1 can increase the *in vivo* stability of Foxp3 by degradation of HIF-1α protein. DTX1 did not bind Foxp3 (Supplementary Fig. 6b), yet DTX1 increased Foxp3 stability (Supplementary Fig. 6c). Unlike Stub1 (ref. 28), DTX1 is not involved in LPS-induced destabilization of Foxp3 protein in Tregs (Supplementary Fig. 6a). DTX1 protected Foxp3 from hypoxia-induced Foxp3 downregulation (Fig. 6b,c) by promoting HIF-1α destabilization. We found that DTX1 bound and promoted HIF-1α degradation in a PHD2-dependent process (Fig. 5e–g), leading to a profound reduction of the HIF-1α protein half-life (Fig. 5b). Notably, DTX1 did not directly promote the ubiquitination of HIF-1α (Supplementary Fig. 7). Instead, DTX1 increased the proline hydroxylation of HIF-1α. The exact molecular processes underlying the binding of DTX1 to HIF-1α and that lead to accelerated HIF-1α degradation are currently being examined. Our results suggest that DTX1-HIF-1α-Foxp3 represents a pathway different from those of inflammatory cytokines in the regulation of the protein stability of Foxp3.

DTX1 belongs to a family of NFAT-dependent E3 ligases that includes Cbl-b, ITCH and GRAIL and that are involved in T-cell anergy^50^. Similar to DTX1, deficiency in ITCH and GRAIL does not affect the development of tTregs and iTregs or expression of Foxp3 (refs 51, 52). The development of tTregs and suppressive function of *Cblb*^*−/−*^ Tregs are also normal^53^. Notably, these E3 ligases regulate the functional integrity of Tregs cells using different mechanisms. *Itch*^*−/−*^ Treg cells secrete excess Th2 cytokines that lead to chronic T-cell activation, skin lesions and antigen-induced airway hypersensitivity^51^. GRAIL knockout in Tregs leads to attenuated suppressive function^52^. Cbl-b-deficiency is accompanied by elevated PI-3 kinase activation, reduced nuclear presence of Foxo3 and Foxo1, and attenuated Foxp3 expression in Tregs^54^. Together, DTX1, Cbl-b, ITCH and GRAIL represent a class of E3 ligases that are not only essential for T-cell anergy, but also are required for the functional integrity of Tregs.

An opposing role of HIF-1α in Foxp3 regulation has also been reported in which HIF-1α transactivates the *Foxp3* promoter and stimulates *Foxp3* expression^55^. In the present study, the stability of Foxp3 was assessed in Treg cells isolated from the spleen and lymph nodes, representing peripheral fully differentiated Tregs. Our study therefore may have excluded the developmental stage of Tregs in which HIF-1α is reported to participate in *Foxp3* expression^55^. However, we found that *Hif1a*^*−*/*−*^ tTregs retained the ability to inhibit colitis (Supplementary Fig. 13), in contrast to the impaired suppressive function of *Hif1a*^*−*/*−*^ tTregs in the same model reported by Clambey *et al.*^55^ We noticed that *Hif1a*^*−*/*−*^ tTregs were less effective than WT tTregs in the readout of colitis score (Fig. 6f), but not in the histology analysis (Supplementary Fig. 13). There was likely difference between *Hif1a*^*−*/*−*^ tTregs and WT tTregs. Further studies will be required to determine the exact window to detect the effects of HIF-1α-knockout in the inhibitory activity of tTregs.

HIF-1α is induced by inflammatory stimuli, cytokines and growth factors even in normoxic conditions^56^. HIF-1α is expressed in T cells that have infiltrated inflammatory tissues^57^. HIF-1α interacts with Foxp3 and promotes Foxp3 degradation^32^. Blocking the binding of HIF-1α to Foxp3 increases Foxp3 levels in Tregs, resulting in enhanced Treg suppressive activities^33^. HIF-1α has been shown to destabilize Foxp3 protein in fully differentiated Tregs in hypoxic or inflammatory states *in vitro*^25^,^28^. This evidence supports the possibility that HIF-1α also mediates the downregulation of Foxp3 in Tregs in a physiological inflammatory environment. We found that DTX1 was able to increase Foxp3 stability through HIF-1α downregulation. In *Dtx1*^*−/−*^ iTregs, HIF-1α induced by TCR stimulation was clearly increased (Fig. 5j), indicating an association between the dysfunctional *Dtx1*^*−/−*^ Tregs and upregulated HIF-1α. In addition, knockout of HIF-1α in *Dtx1*^*−/−*^ tTreg and *Dtx1*^*−/−*^ iTreg cells restored Foxp3 expression and its suppressive activity *in vivo* (Fig. 6e–h and Supplementary Figs 11–14). Our results therefore suggest that the function of Tregs *in vivo* requires factors like DTX1 to sustain stable expression of Foxp3 under hypoxic or inflammatory conditions. DTX1 represents another level of control of Treg function by regulating the protein stability of Foxp3 against the destabilizing effect of HIF-1α *in vivo*. Notably, Tregs are present at high frequency in tumour tissues and are associated with suppression of effector T cells within the tumour microenviroment^58^,^59^. Tumours contain hypoxic regions, with increased HIF-1α protein levels^46^. Given the ability of HIF-1α protein to degrade Foxp3 (refs 32, 33), we propose that DTX1 may also contribute to the stability of tumour-infiltrated Tregs by antagonizing HIF-1α.

Tregs are one of the major components in immune tolerance maintenance^3^,^4^,^5^,^6^,^7^. Administration of Tregs to restore immune tolerance has been explored as a potential therapeutic approach in transplantation, autoimmune diseases and allergy^18^,^19^,^20^,^21^. The present study illustrates a new level of regulation on the stability of Tregs by antagonizing HIF-1α. It may be noted that DTX1 is unlikely the only protein that participates in the downregulation of HIF-1α. Given the diverse proinflammatory stimuli Tregs may encounter in their lifespan, DTX1 may coordinate with several stabilizing factors to confer full protection on Foxp3 protein expression in Tregs. Identification of these stabilizing factors and further characterization of the molecular mechanism underlying the Foxp3-stabilizing effect of DTX1 will help provide a full molecular picture on the physiological regulation of Tregs, as well as establish the basis for the application of Tregs in immunotherapy.

## Methods

### Reagents

Mouse deltex1 cDNA^36^ was a gift from Dr Hideyuki Okano (Keiko University, Tokyo, Japan). HIF-1α and HIF-1α (ΔODD) were obtained from Dr Kou-Juey Wu (National Yang-Ming University, Taipei, Taiwan). Polyclonal anti-DTX1 antibody was generated by immunizing rabbits with recombinant DTX1. OVA, alum, anti-FLAG (M2, 1;1,000) and the periodic acid–Schiff (PAS) staining system were purchased from Sigma. The antibodies against glyceraldehyde 3-phosphate dehydrogenase (6C5, 1:5,000) and His (H-3, 1:1,000) were obtained from Santa Cruz Biotech. Fluorescein isothiocyanate (FITC)-labelled streptavidin was purchased from Invitrogen/Caltag. Anti-IFN-γ (XMG1.2, 1:100), anti-IL-17 (18H10, 1:100), anti-CD103 (M290, 1:100), anti-HIF-1α (54/HIF-1α, 1:1,000), anti-VHL (Ig32, 1:500) and Cytometric Bead Array were obtained from BD-Bioscience. Anti-HIF-1α (1:1,000) was purchased from Cayman. FITC- or PE-conjugated anti-CD25 (PC61.5.3, 1:100), anti-LAG3 (C9B7W, 1;100), anti-ROR-γt (B2D, 1:2,000), anti-Foxp3 (eBio7979, 1:500), FITC-conjugated anti-Foxp3 (FJK-16s, 1:100), anti-CD44 (IM7, 1:100), anti-CD45.1 (A20, 1:100) and PE-conjugated anti-FR4 (12A5, 1:100) were purchased from eBioscience. FITC- or PE-conjugated anti-CD4 (GK1.5, 1:100), PE-conjugated anti-CD152 (CTLA-4; UC10-4B9, 1:100), PE-conjugated anti-GITR (YGITR765, 1:100), anti-CD62L (Mel-14, 1:100), anti-CD45.2 (104, 1:100), anti-CCR4 (2G12, 1:100), anti-CCR5 (HM-CCR5, 1:100) and anti-CCR6 (29-2L17, 1:100) were obtained from BioLegend. Anti-Myc (9B11, 1:1,000) and anti-hydroxyl HIF-1α (Pro564, 1:1,000) were purchased from Cell Signaling. Anti-PHD2 (NB100-137, 1:2,000) was obtained from Novus. Biotinylated anti-TGF-β1 (1:200) and anti-IL-12 p35 (27537) were obtained from R&D. Anti-β-tubulin (1:2,000) was obtained from Upstate. Anti-lamin B1 (1:1,000) was purchased from Zymed. Horseradish peroxidase-conjugated secondary antibodies and ECL were obtained from Amersham Bioscience. The reverse transcription–PCR primers were as follows: *Dtx1*: forward 5′-CAAAGCCATCTACGGGGA-3′, reverse 5′-TGGCCATGGCCTCAGAAAC-3′; *Foxp3*: forward 5′-AGCTGCAGCTGCCTACAG-3′, reverse 5′-CCAGCAGTGGGTAGGATC-3′; *Ccr4*: forward 5′-ATCCTGAAGGACTTCAAGCTCCA-3′, reverse 5′-AGGTCTGTGCAAGATCGTTTCATGG-3′; *Ccr6*: forward 5′-CCTCACATTCTTAGGACTGGAGC-3′, reverse 5′-GGCAATCAGAGCTCTCGGA-3′; *Ccr7*: forward 5′-ATGCTGGCTATGAGTTTC-3′, reverse 5′-GCTGCTATTGGTGATGTT-3′; *Ebi3*: forward 5′-AGCAGCAGCCTCCTAGCCT-3′, reverse 5′-ACGCCTTCCGGAGGGTC-3′; *Foxo1*: forward 5′-ACGAACTCGGAGGCTCCTTAGACAC-3′, reverse 5′-GACTGGAGGTGGTCGAGTTGGACTG-3′; *Foxo3a*, forward 5′-ATGGGCCACGATAAGT-3′, reverse 5′-CAGTAACAGTCCGCCT-3′; *Gzmb*: forward 5′-CTCCACGTGCTTTCACCAAA-3′, reverse 5′-AGGATCCATGTTGCTTCTGTAGTTAG-3′; *Ikzf4*: forward 5′-CCAAGTCCCTGAGTGGTTGT-3′, reverse 5′-TTATCCAGGAGCCGTTCATC-3′.

### Mice

The *Dtx1*^*f/f*^ mice and *Dtx1*^*−/−*^ mice (*Dtx1*^*f/f*^*EIIa-Cre,* mice with systemic deletion of *Dtx1*) were previously described^40^. *Rag1*^*−*/*−*^ mice, NOD/ShiLt-Tg(Foxp3^EGFP/Cre^)1Jbs/J mice (also known as NOD.Foxp3-Cre)^41^ and B6.129-*Hif1a*^*tm3Rsjo*^/J (*Hif1a*^*f/f*^)^60^ were obtained from Jackson Laboratories. NOD.Foxp3-Cre mice were back-crossed to C57BL6 mice for at least eight generations. *Foxp3*^*RFP-Cre*^ transgenic ((C57BL/6-Tg(Foxp3-RFP,-Cre)1Narl) mice were generated by National Laboratory Animal Center, Taiwan, with the construct similar to that in *Foxp3*^*EGFP-Cre*^ mouse. No difference was observed between *Foxp3*^*RFP-Cre*^ mouse and *Foxp3*^*GFP-Cre*^ mouse in Treg-specific knockout of DTX1, and *Foxp3*^*Cre*^ mouse was used to represent either one. *Cd4-Cre* mice were obtained from Taconic Farms. All mice used were 8–10 weeks old. For colitis experiments, only female donors and recipients mice were used. For other *in vivo* experiments, sex-matched male and female mice were used. Both male and female mice were used for *in vitro* experiments. All mouse experiments were conducted with the approval of the Experimental Animal Committee, Academia Sinica.

### Cell culture and transfection

Jurkat cells (clone E6-1, TIB-152) were obtained from American Type Culture Collection. DO11.10 cells were I-A^d^-restricted, OVA-specific T-cell hybridomas^61^. T cells were cultured in RPMI media with 10% fetal calf serum (both from GIBCO), 10 mM glutamine, 100 U ml^*−*1^ penicillin, 100 U ml^*−*1^ streptomycin and 2 × 10^−5^ M 2 mercaptoethanol (ME). DMEM was used instead of RPMI in the culture of 293T cells.

For the transfection of 293T cells, 1 × 10^6^ 293T cells were seeded overnight. Plasmid DNA (1–4 μg) and OmicsFect *in vitro* Transfection Reagent (2–8 μl, in a ratio of 1 μg DNA to 2 μl reagent; Omics Biotechnology) were diluted separately in 250 μl serum-free DMEM medium. The diluents were mixed slowly, set at room temperature for 15 min and were then added to 293T cells. 293T cells were analysed 24 h later. Transfection of Jurkat and DO11.10 T cells was conducted by electroporation on a Neon Transfection System (MP-100 Microporator, Invitrogen). A total of 1 × 10^7^ T cells were washed twice with PBS and resuspended in 100 μl resuspension R buffer (Invitrogen). A measure of 500 pmole short interfering RNA or 10 μg plasmid DNA were added to cells, and T cells were electroporated at 1,700 V, 30 ms, 1 pulse. T cells were then resuspended in 10 ml antibiotic-free complete RPMI medium, and cultured for 24–72 h before further analysis.

For retroviral infection of T cells, Foxp3 or DTX1 were subcloned into pGCIRES-YFP, a gift from Dr Gina Costa (Stanford University). Retroviruses were produced using Phoenix-Eco cells (gifts of Dr Garry P Nolan, Stanford University)^39^. DO11.10 T cells were mixed with retroviruses-containing supernatants with polybrene (1 mg ml^−1^), and spin-infected at 1,900 r.p.m. for 45 min at 30 °C. Two days after infection, YFP-expressing DO11.10 cells were isolated by sorting on FACSVantage SE (Becton Dickinson).

### Cell lysates and immunoblots

For the preparation of whole-cell lysates, cells were lysed by whole-cell extract buffer (25 mM HEPES, pH 7.7, 300 mM NaCl, 0.1% Triton X-100, 1.5 mM MgCl_2_, 0.2 mM EDTA, 0.1 mM Na_3_VO_4_, 50 mM NaF, 0.5 mM dithiothreitol and 10% glycerol) on ice for 30 min. Debris was removed by centrifuge on a Eppendoff microfuge (13,200 r.p.m., 30 min, 4 °C), and protein concentrations in the supernatants quantitated by Bio-Rad protein assay.

For immunoprecipitation, whole-cell lysates were incubated with 1 μg specific antibody overnight at 4 °C. This was followed by incubation with Protein G Mag Sepharose (10 μl; GE Healthcare) for 2 h at 4 °C. The beads were washed five times and analysed by SDS–polyacrylamide gel electrophoresis (SDS–PAGE).

For immunoblots, lysates or immunoprecipitates resolved by SDS–PAGE were transferred to polyvinylidene difluoride membrane (Millipore) with transfer buffer (30 mM Tris, 250 mM glycine, 1 mM EDTA, 20% methanol) at 4 °C for 2 h at 400 mA. Membranes were blocked with 5% non-fat milk in 0.1% TBST (10 mM Tris-HCl pH 8.0, 150 mM NaCl, 0.1% Tween-20) at room temperature for 1 h. The membrane was then incubated with primary antibodies overnight at 4 °C. After washing, the membrane was incubated with horseradish peroxidase-conjugated secondary antibodies in blocking buffer for 1.5 h at room temperature. After washing three times, membranes were developed using ECL western blot detection reagents (GE Healthcare), and signals detected by X-ray film. Immunoblot images have been cropped for presentation. Full-size images are presented in Supplementary Figs 15–17.

### Regulatory T cells

Treg cells from splenocytes were purified using a magnetic-activated cell sorting (MACS) CD4^+^CD25^+^ Regulatory T Cell Isolation Kit (Miltenyi Biotech) or by fluorescence-activated cell sorting (FACS). The intracellular staining of Foxp3 was conducted on paraformaldehyde-fixed splenocytes pre-labelled for CD4 and CD25. Cells were then permeabilized with 0.2% saponin and stained with FITC-conjugated anti-Foxp3 (1:100, eBioscience). IL-10 and TGF-β levels were measured in supernatants from tTreg cells stimulated with anti-CD3 (5 μg ml^*−*1^), anti-CD28 (2 μg ml^*−*1^) and IL-2 (50 U ml^*−*1^) for 3 days by a mouse IL-10 DuoSet ELISA System (R&D) and a TGF-β OptEIA Set (BD Bioscience), respectively. For the staining of surface TGF-β1, CD4^+^CD25^+^ T cells were stimulated with plate-bound anti-CD3/anti-CD28 and IL-2 (50 U ml^*−*1^) for 96 h, fixed with 2% paraformaldehyde and labelled sequentially with biotinylated anti-TGF-β1 (1:100) and FITC-streptavidin.

Induced Treg (iTreg) cells were generated from CD4^+^CD25^*−*^ T cells by treating with immobilized anti-CD3 (5 μg ml^*−*1^) and anti-CD28 (1 μg ml^*−*1^) in the presence of TGF-β (5 ng ml^*−*1^) for 5–7 days. CD4^+^CD25^+^ cells were isolated by MACS at day 5 and used for *in vitro* and *in vivo* suppression analysis. For expression of Treg-associated molecules, iTreg cells after 6-day treatment were first stained with antibodies specific for CD103, CTLA-4, FR4, GITR, LAG3 or TGF-β, followed by secondary antibody. Cells were then permeabilized, stained with FITC-conjugated anti-Foxp3 and the expression of each marker in the Foxp3^+^ population was analysed by FACS.

### *In vitro* suppressive assay of Tregs

CD4^+^CD25^*−*^ cells were placed into round-bottom 96-well plates at 1 × 10^5^ cells per well; each well also contained anti-CD3 (1 μg ml^*−*1^), 3 × 10^5^ γ-irradiated T cell-depleted splenic cells, and 1.25, 2.5, 5 or 10 × 10^4^ CD4^+^CD25^+^ Treg cells. Cells were incubated for 72 h. [^3^H]thymidine was then added, and cells were harvested 8 h later for thymidine incorporation determination. IFN-γ production was measured 40 h after TCR stimulation by ELISA.

### Induction of colitis in Rag1^*−*/*−*^ mice

CD4^+^CD25^*−*^ T cells (4 × 10^5^) from female mice were administered by intraperitoneal injection into female RAG1^*−*/*−*^ mice (8- to 10-week-old) with or without 1 × 10^5^ tTregs from female mice. Colitis was assessed weekly. The score for wasting was as follows: 0, no wasting; 1 for up to 10% initial body weight loss and 2 for >10% initial body weight loss. Rectal prolapse was scored as 0 for absence and 1 for presence. Diarrhoea was scored as 0 for absence, 1 for soft stool and 2 for watery/bloody stool. Hunching/bristled fur/skin lesions were scored as 0 for absence and 1 for presence. At 7 weeks after T-cell transfer, mice were killed and blood was removed by cardiac perfusion with PBS. Colons were removed, cleaned by PBS, fixed in 4% paraformaldehyde and then embedded in paraffin. Sections were stained with haematoxylin and eosin or PAS staining system.

### Allergen-induced airway inflammation

C57/BL6 mice (8- to 10-week-old) were intravenously injected with or without 2.5 × 10^6^ iTreg cells or PBS on day 0, and sensitized with two intraperitoneal doses of 50 μg OVA in 2 mg alum solution^62^ on day 1 and day 14. Mice were challenged daily for 30 min with OVA by placing them in a chamber where an aerosolized solution of 1% OVA in saline (0.9% NaCl) was generated by a Nebulizer 646 in aerosol therapy system (DeVilbliss) from days 21 to 24. On day 25, mice were killed, perfused with PBS and lungs were fixed with 4% PFA followed by staining with haematoxylin and eosin and PAS.

### *In vitro* ubiquitination assay

Bovine ubiquitin, rabbit rhUBE1 (E1) and UbcH5c (E2) were purchased from BostonBiodem. The *in vitro* ubiquitination reaction mixture contained ubiquitin (1 μg), E1 (0.2 μg), E2 (0.2 μg), His-Deltex1 (0.2 μg), His-Deltex1ΔRF (0.2 μg), GST-HIF-1α (0.5 μg) or GST-MEKK1(C) (0.5 μg), as indicated, in 30 μl of reaction buffer (25 mM Tris, pH 7.5, 50 mM NaCl, 10 mM MgCl_2_, 2 mM ATP, 0.5 mM dithiothreitol). The reaction mixture was incubated at 30 °C for 1 h. Ubiquitin and ubiquitin-associated proteins were pulled down from the reaction mixture by incubation with 1 μg of anti-ubiquitin (Millipore) overnight at 4 °C, followed by incubation with Protein G Mag Sepharose (20 μl) at 4 °C for additional 2 h. Mag Sepharose was then washed five times with reaction buffer without ATP, and resolved on SDS–PAGE. The amount of ubiquitin-associated protein was determined by blotting with anti-GST (1:5,000, GE).

## Author contributions

M.-Z.L. conceived of the idea, designed and supervised the experiments, and wrote the manuscript, H.-W.H., T.-S.H. and W.-H.L. performed *in vitro* and *in vivo* Treg analysis, W.-C.H., T.-F.C. and Y.-J.W. performed selected immunoblots, S.-T.J. generated *Foxp3*^*RFP-Cre*^ transgenic mice.

## Additional information

**How to cite this article:** Hsiao, H.-W. *et al.* Deltex1 antagonizes HIF-1α and sustains the stability of regulatory T cells *in vivo*. *Nat. Commun.* 6:6353 doi: 10.1038/ncomms7353 (2015).

## Supplementary Material

Supplementary Information Supplementary Figures 1-17.
