# Supplementary material for: Deltex1 antagonizes HIF-1α and sustains the stability of regulatory T cells in vivo
Source: Nat Commun. 2015 Feb 19;6:6353. doi: 10.1038/ncomms7353 (PMC4346631; doi:10.1038/ncomms7353)
Supplement: Supplementary Information — Supplementary Figures 1-17. [file ncomms7353-s1.pdf]

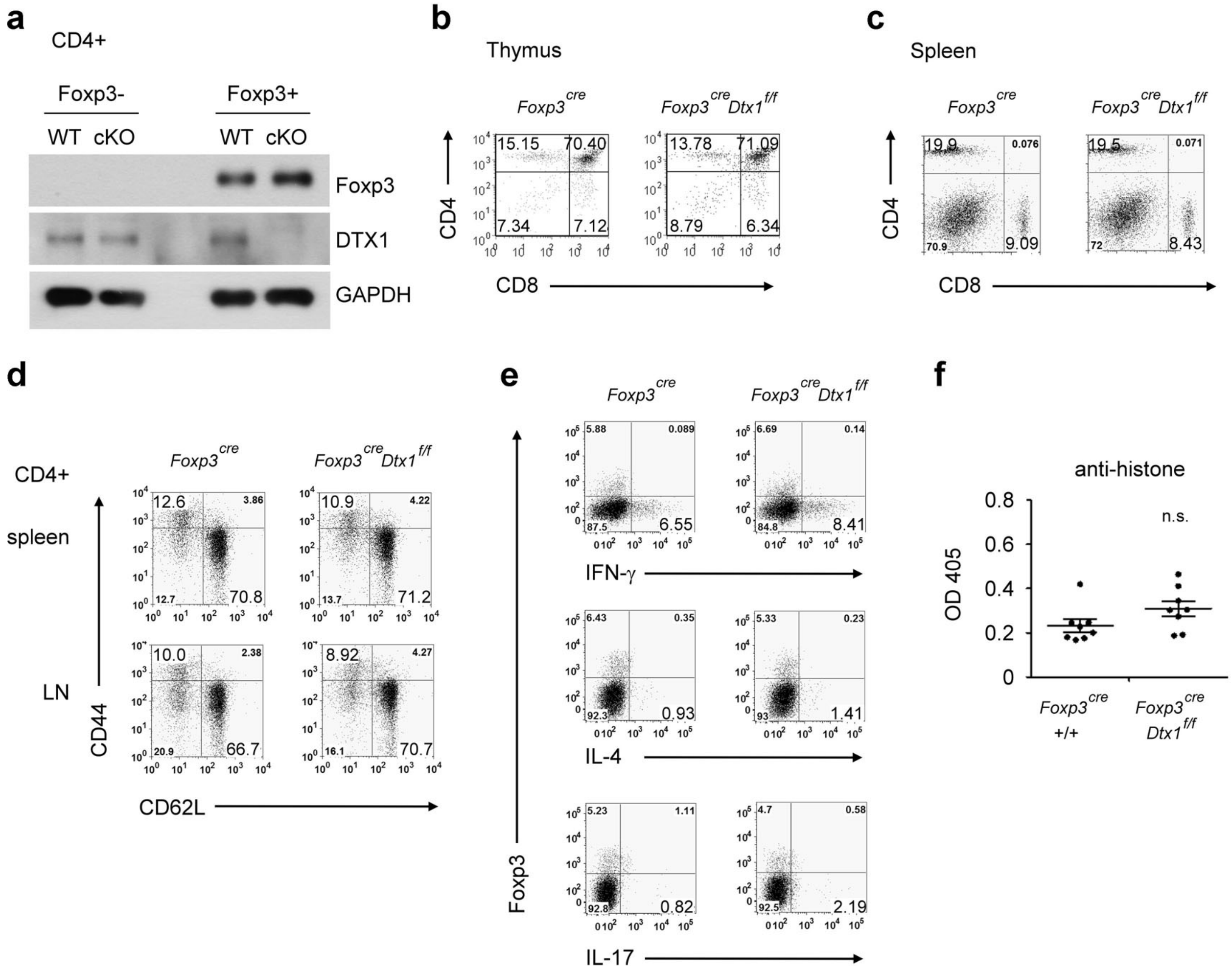

Supplementary Figure 1. Regulatory T cell-specific deficiency in DTX1 does not affect T cell development *in vivo*. (**a**) Knockdown of *Dtx1* in Tregs. CD4$^+$Foxp3$^-$ and CD4$^+$Fopx3$^+$ T cells were purified from spleens of *Foxp3*$^{RFP\text{-}Cre}$ (*Foxp3*^{Cre}, WT) and *Foxp3*$^{RFP\text{-}Cre}$*Dtx1*$^{f/f}$ (*Foxp3*^{Cre}*Dtx1*$^{f/f}$, cKO) mice based on RFP expression. The levels of Foxp3 and DTX1 were determined by immunoblot. (**b, c**) T cell development was not affected by Treg-conditional knockout of *Dtx1*. Thymocytes (b) and splenocytes (c) from *Foxp3*^{Cre} (WT) and *Foxp3*^{Cre}*Dtx1*$^{f/f}$ (cKO) mice were stained for expression of CD4 and CD8. (**d**) Normal naïve and memory cell ratios in T cells from *Foxp3*^{Cre}*Dtx1*$^{f/f}$ mice. CD4$^+$ T cells in spleen and lymph nodes from WT and cKO mice were gated, and CD44^{hi}CD62L^{lo} and CD44^{lo}CD62L^{hi} populations were quantified. (**e**) Weak increase in the production of IFN-γ and IL-17 in *Foxp3*^{Cre}*Dtx1*$^{f/f}$ T cells. Freshly isolated splenic T cells from WT (*Foxp3*^{cre}) and DTX1-cKO (*Foxp3*^{Cre}*Dtx1*$^{f/f}$) mice were stimulated for 5 h *in vitro* with PMA (50 ng ml^{-1}) and ionomycin (500 ng ml^{-1}) in the presence of monensin (2 nM), and expression of IFN-γ, IL-4, and IL17 were measured by intracellular staining. (**f**) Comparable anti-histone antibody titer between WT and cKO mice. Sera from *Foxp3*^{Cre} and *Foxp3*^{Cre}*Dtx1*$^{f/f}$ mice (n=8) older than 6 months were examined for anti-histone antibodies.

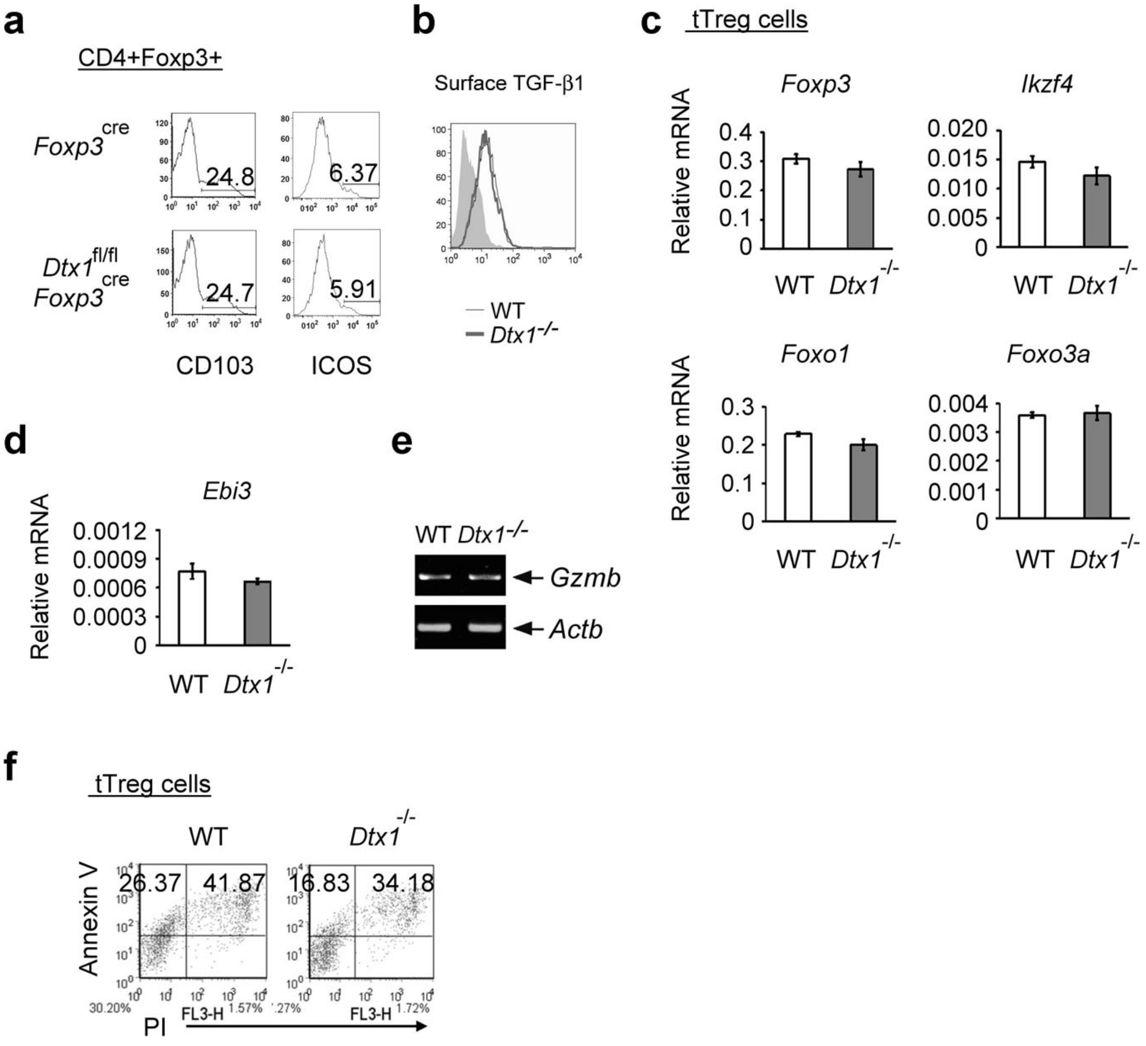

Supplementary Figure 2. The expression of Treg-associated molecules is not affected by deficiency in DTX1 in Tregs. (**a**) Normal expression of CD103 and ICOS in DTX1-cKO tTregs. $CD4^+$ T cells from WT (*Foxp3*$^{GFP-cre}$) and DTX1-cKO (*Foxp3*$^{GFP-cre}$*Dtx1*$^{f/f}$) were stained for surface expression of CD103 and ICOS, and the $CD4^+GFP^+$ population was analyzed by flow cytometry. (**b**) Surface TGF-β expression in tTregs is not affected by DTX1-deficiency. $CD4^+CD25^+$ cells (tTregs) from WT and *Dtx1*$^{-/-}$ mice were stimulated with anti-CD3 and anti-CD28 in the presence of IL-2 for 96 h, and the surface TGF-β expression was assessed by flow cytometry. (**c, d, e**) Comparable expression of *Foxp3*, *Ikzf4*, *Foxo1*, *Foxo3*, *Ebi3*, and *Gzmb* between WT and *Dtx1*$^{-/-}$ tTregs. RNA was purified from WT and *Dtx1*$^{-/-}$ tTregs, and transcripts of *Foxp3*, *Ikzf4*, *Foxo1*, *Foxo3* (c), *Ebi3* (d), *Gzmb* (e) were determined by quantitative RT-PCR (c, d) or RT-PCR (e). Values in (c, d) were normalized against *Gapdh*. (**f**) Activation-induced cell death in Tregs is not affected by DTX1-deficiency. WT and *Dtx1*$^{-/-}$ tTregs were stimulated with anti-CD3 (5 μg ml^{-1}) for 3 days. Cell death was determined by staining with Annexin V and PI, and was defined by the sum of Annexin V^+ and PI^+ populations.

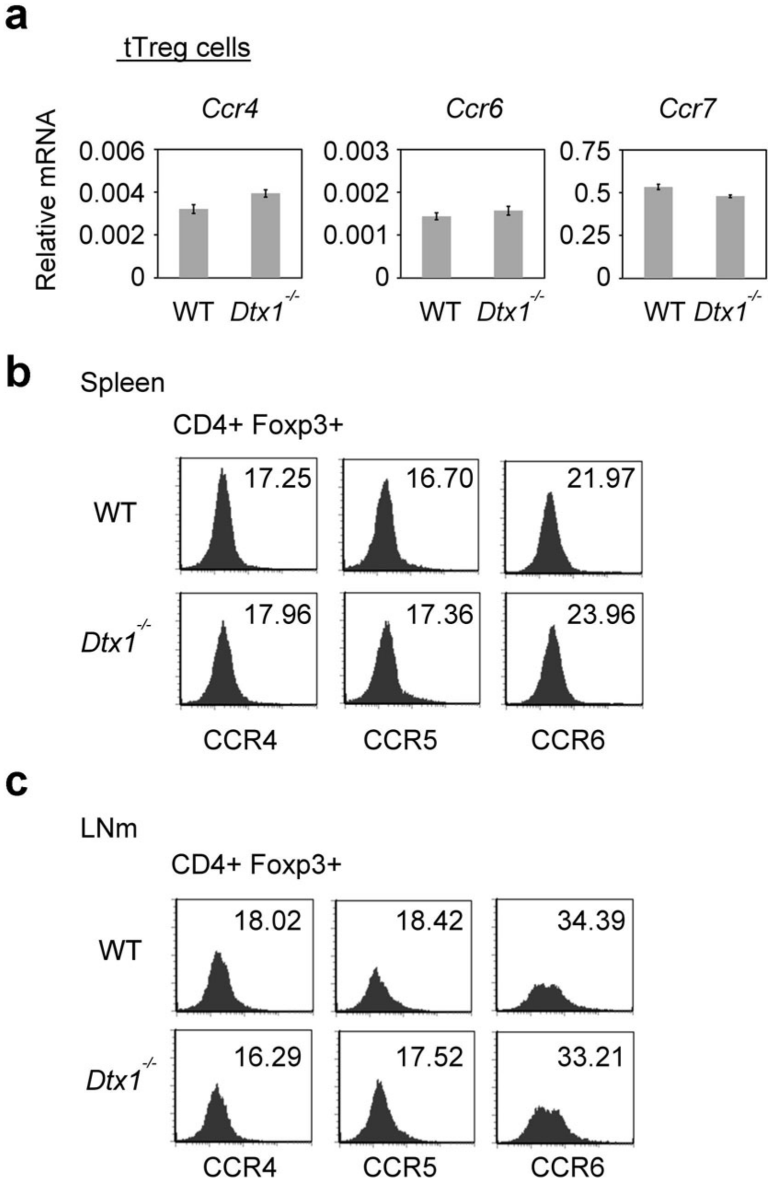

Supplementary Figure 3. *Dtx1*-deficiency does not affect the expression of chemokine receptors in Treg cells. (**a**) RNA from WT and *Dtx1*$^{-/-}$ tTregs was isolated, and the transcripts of *Ccr4*, *Ccr6*, and *Ccr7* were determined by quantitative RT-PCR. Values were normalized against *Gapdh* transcript. (**b, c**) Splenocytes (**b**) and mesentery lymph node cells (**c**) were stained with anti-CCR, followed by intracellular staining with anti-Foxp3, and the surface expression of CCR4, CCR5, and CCR6 of the gated CD4$^+$Foxp3$^+$ population were quantitated by flow cytometry. Numbers indicate mean fluorescence intensity.

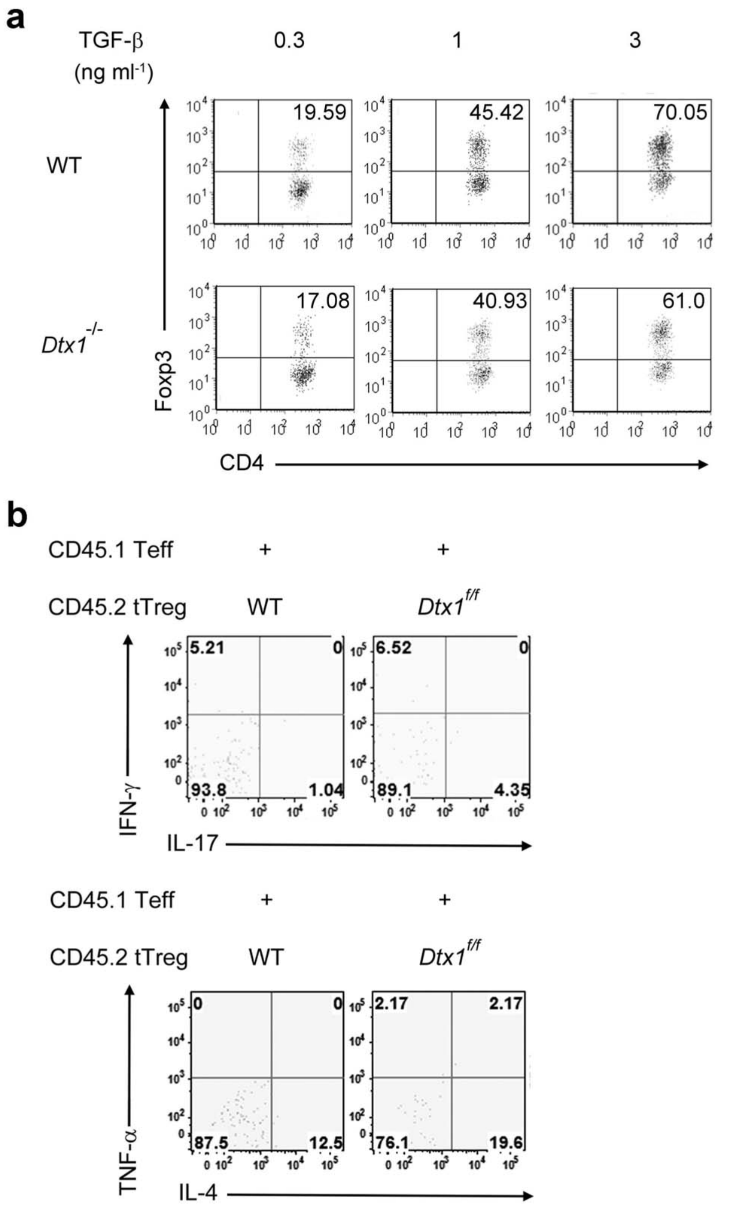

Supplementary Figure 4. Suboptimal TGF-β does not impair *Dtx1*$^{-/-}$ iTreg differentiation, and small increase in the expression of IFN-γ, IL-17, TNF-α, and IL-4 in transferred *Dtx1*$^{-/-}$ T cells. (**a**) Suboptimal differentiation of iTreg is not affected by DTX1-deficiency. $CD4^+CD25^-$ T cells from WT and *Dtx1*$^{-/-}$ mice were treated with plate-bound anti-CD3 (2 μg ml^{-1}) and anti-CD28 (1 μg ml^{-1}) in the presence of TGF-β at the indicated concentrations and IL-2 (20 ng ml^{-1}) for 4 days, and Foxp3 expression was assessed by intracellular staining. (**b**) *Rag1*$^{-/-}$ mice were transferred with $CD45.1^+$ $CD4^+CD25^-$ T cells, and 1 × 10^5 $CD45.2^+$ WT (*Foxp3*^{Cre}) or *Foxp3*^{Cre}*Dtx1*$^{f/f}$ tTreg cells. $CD4^+CD45.1^-$ T cells were isolated one week later, and were stimulated with PMA/ionomycin in the presence of monensin for 5 h. The expression of IFN-γ, IL-17, TNF-α, and IL-4 were measured by intracellular staining. Data is representative of 3 mice.

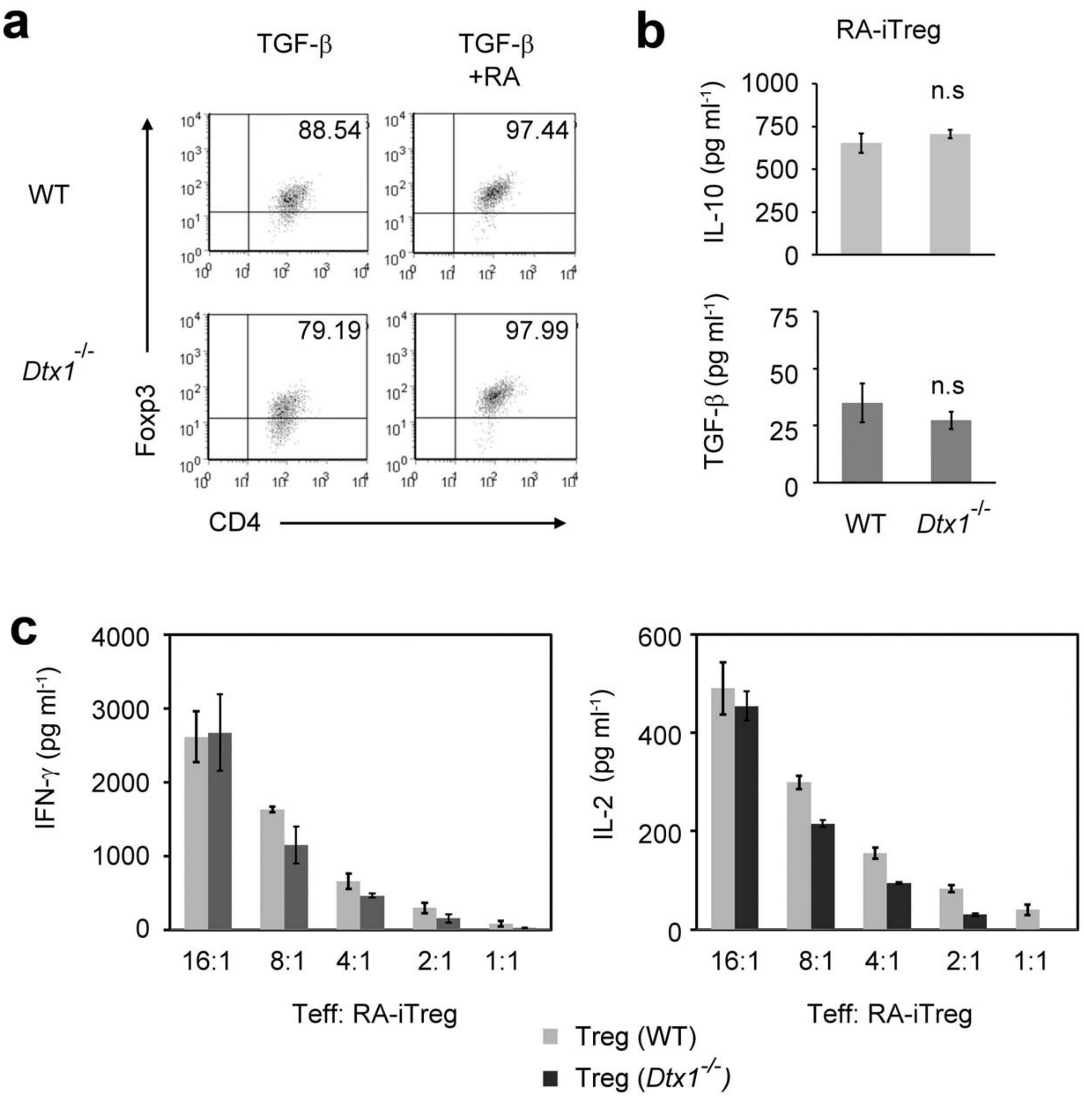

Supplementary Figure 5. Normal responses of *Dtx1*$^{-/-}$ T cells to retinoic acid for iTreg differentiation and function. WT and *Dtx1*$^{-/-}$ $CD4^+CD25^-$ T cells were allowed to differentiate into iTregs in the absence or presence of retinoic acid (10 nM) for 4 days, and the fraction of $Foxp3^+$ cells was determined (**a**). The iTregs generated with or without retinoic acid treatment were stimulated with anti-CD3 and anti-CD28 for 3 days, and the secretion of IL-10 and TGF-β quantitated (**b**). Effector ($CD4^+CD25^-$) T cells incubated with retinoic acid co-generated WT and *Dtx1*$^{-/-}$ iTreg cells at the indicated ratio in the presence of irradiated B cells and anti-CD3. The production of IL-2 and IFN-γ was measured 48 h and 72 h after stimulation (**c**).

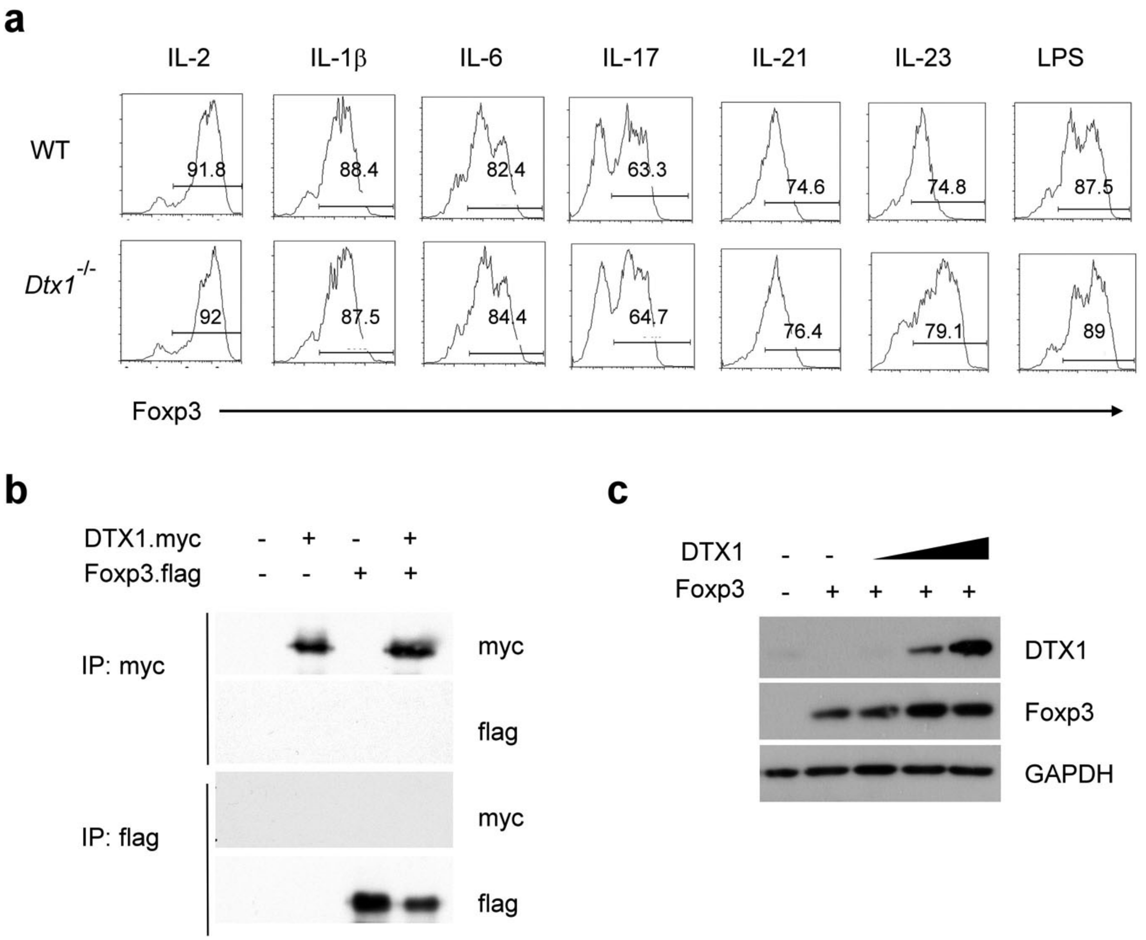

Supplementary Figure 6. DTX1 increases Foxp3 expression independent of inflammatory cytokines and direct interaction. (**a**) Inflammatory cytokine-induced Foxp3 downregulation is not affected by DTX1 deficiency in tTregs. WT and *Dtx1*$^{-/-}$ tTregs were treated with IL-2 (20 ng ml^{-1}), IL-1β (50 ng ml^{-1}), IL-6 (30 ng ml^{-1}), IL-17 (20 ng ml^{-1}), IL-21 (20 ng ml^{-1}), IL-23 (20 ng ml^{-1}), or LPS (100 ng ml^{-1}), and Foxp3 contents were determined by intracellular staining and flow cytometry 72 h later. (**b**) DTX1 does not interact with Foxp3. DTX1-Myc and Foxp3-Flag were transfected into Jurkat cells, and cell lysates prepared 24 h later. Cell lysates were immunoprecipitated with anti-Myc or anti-Flag, and the presence of DTX1-Myc and Foxp3-Flag in precipitates was determined. (**c**) Increased Foxp3 protein levels by DTX1 co-expression. DTX1 (0.2, 0.5, or 1 μg) and Foxp3 were transfected into 293T cells, and cellular levels of Foxp3 were determined 48 h after transfection.

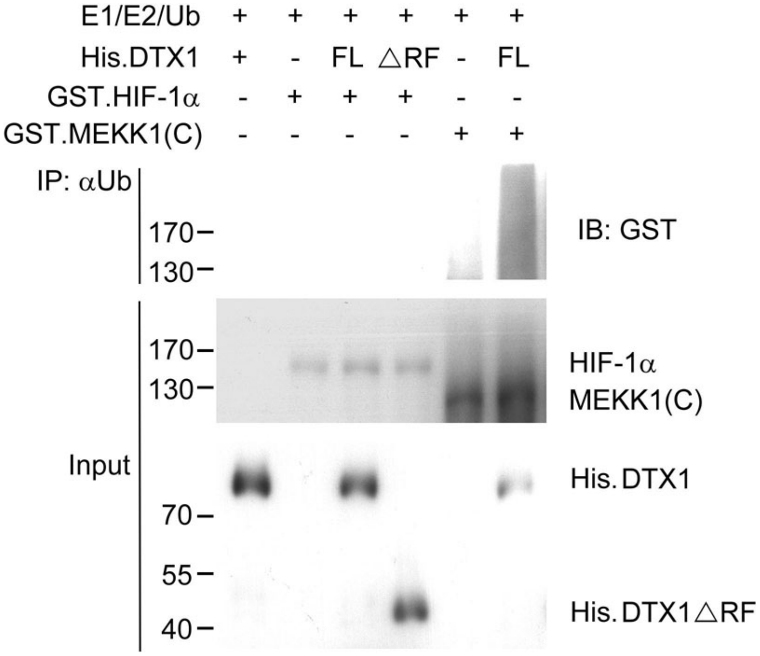

Supplementary Figure 7. DTX1 does not ubiquitinate HIF-1α *in vitro*. *In vitro* ubiquitination assays were conducted in reaction mixtures containing ubiquitin, E1, E2, His-DTX1, HIS-DTX1(ΔRF), GST-HIF-1α, or GST-MEKK1(C), as indicated. Reactions proceeded at 30°C for 1 h. Anti-ubiquitin and protein-G were used to pull down ubiquitinated protein, separated on SDS-PAGE, and blotted with anti-GST to detect HIF-1α and MEKK1 (upper panel). The input is shown in the bottom panel.

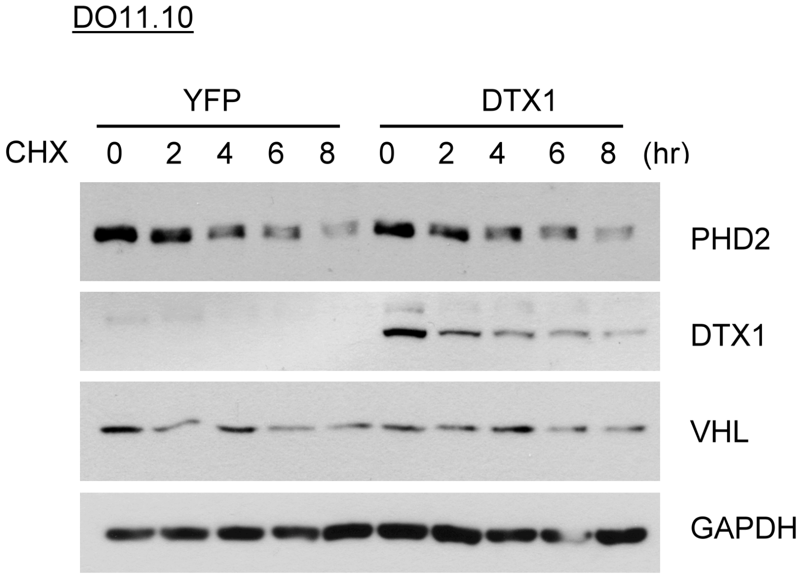

Supplementary Figure 8. DTX1 does not affect the protein stability of PHD2 and VHL. Control and DTX1-expressing DO11.10 T cells were treated with cycloheximide (CHX), and cell lysates were prepared at the indicated times. The amounts of PHD2 and VHL were determined.

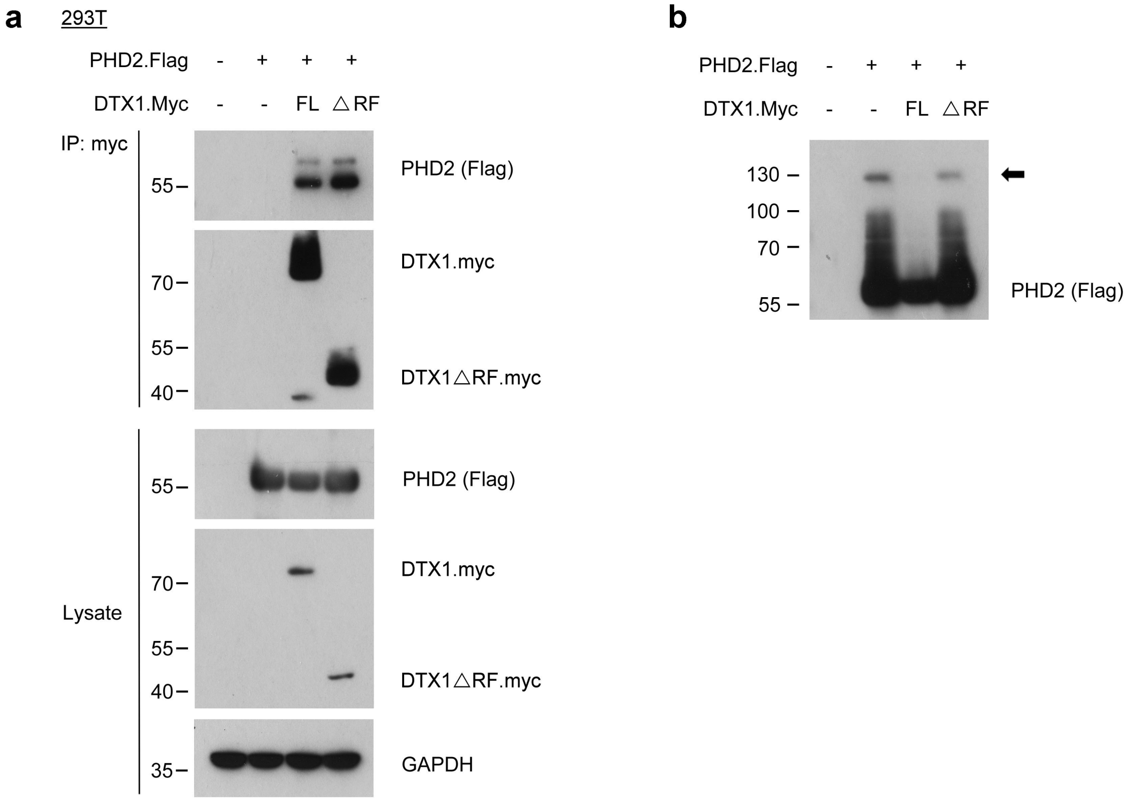

Supplementary Figure 9. DTX1 binds PHD2 and prevents PHD2 oligomerization. (**a**) Interaction between DTX1 and PHD2. 293T cells were transfected with DTX1-Myc, DTX1ΔRF-Myc, or PHD2, as indicated. Cell lysates were prepared 24 h after transfection, and were precipitated with anti-Myc. The contents of PHD2 and DTX1 in the immunoprecipitates and lysates were determined. (**b**) DTX1, but not DTX1ΔRF, inhibits PHD2 oliogmerization. Immunoblot in (a) was exposed longer to detect modification and trimerization of PHD2 (indicated by arrow).

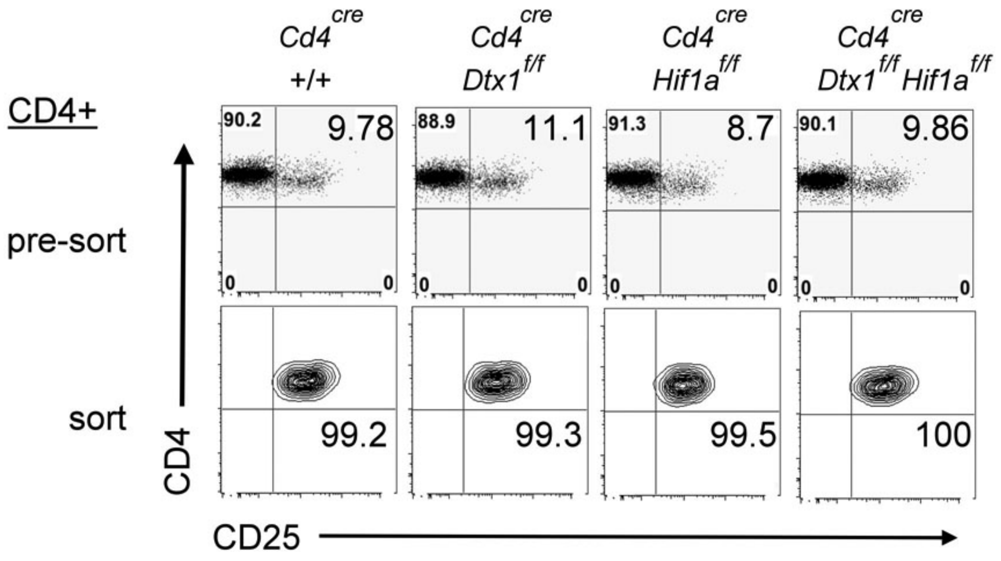

Supplementary Figure 10. Similar tTreg population in mice deficient in *Dtx1*, *Hif1a*, or both, and the purity of tTregs used in the present study. Upper panel, frequency of $CD4^{+}CD25^{+}$ T cells in $CD4^{+}$ splenic T cells from *Cd4*^{Cre}, *Cd4*^{Cre} × *Dtx1*$^{f/f}$, *Cd4*^{Cre} × *Hif1a*$^{f/f}$, and *Cd4*^{Cre} × *Dtx1*$^{f/f}$*Hif1a*$^{f/f}$ mice. Lower panel, the purity of tTreg cells isolated by sorting for the experiments shown in Fig. 6e.

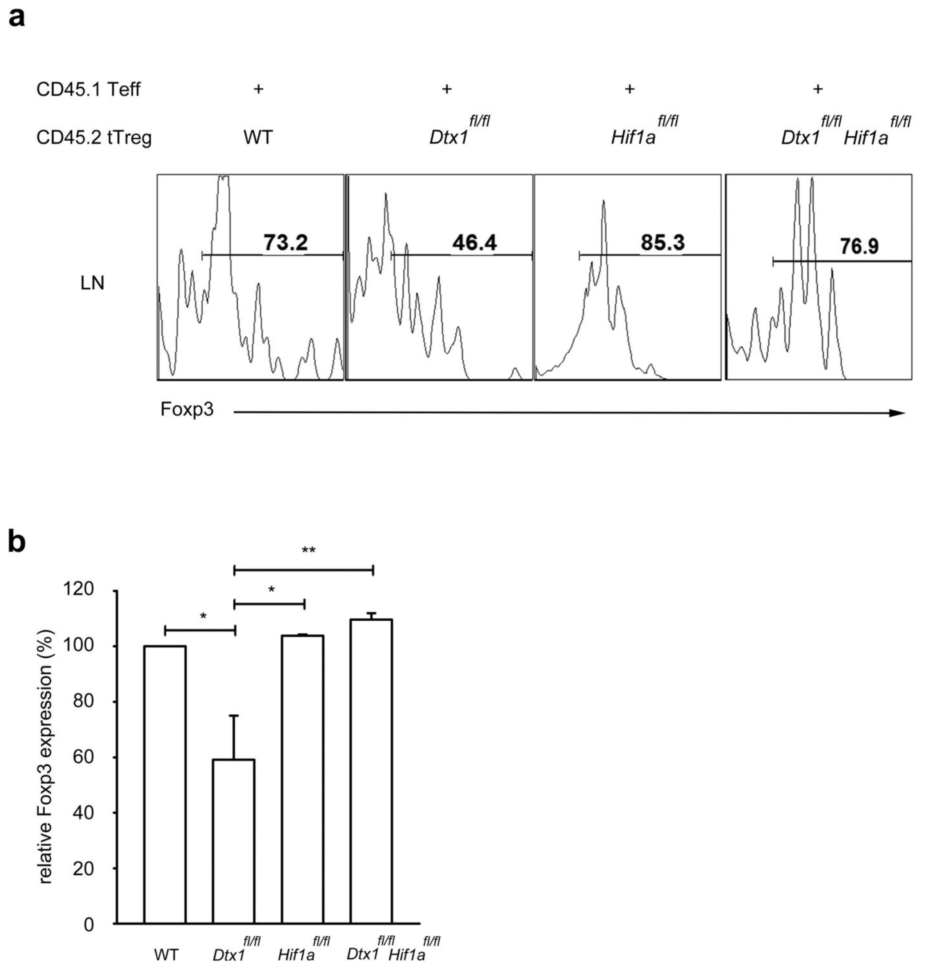

Supplementary Figure 11. Reduced Foxp3^{+} population in transferred *Dtx1$^{-/-}$* tTregs is restored by HIF-1α-deficiency. CD45.2^{+} WT (*Dtx1$^{f/f}$*), *Foxp3^{Cre}Dtx1$^{f/f}$*, *Foxp3^{Cre}Hif1a$^{f/f}$*, or *Foxp3^{Cre}Dtx1$^{f/f}$Hif1a$^{f/f}$* RFP^{+} (tTreg) cells were co-transferred with CD45.1^{+} CD4^{+}CD25^{-} T cells into *Rag1$^{-/-}$* mice. Lymph nodes were isolated one week later, and the frequency of Foxp3^{+} cells in the CD4^{+}CD45.2^{+} T cell population was determined. Bottom panel, average of lymph node Foxp3^{+} cells from 3 mice in each group. The percentage of +/+ was set as 100%.

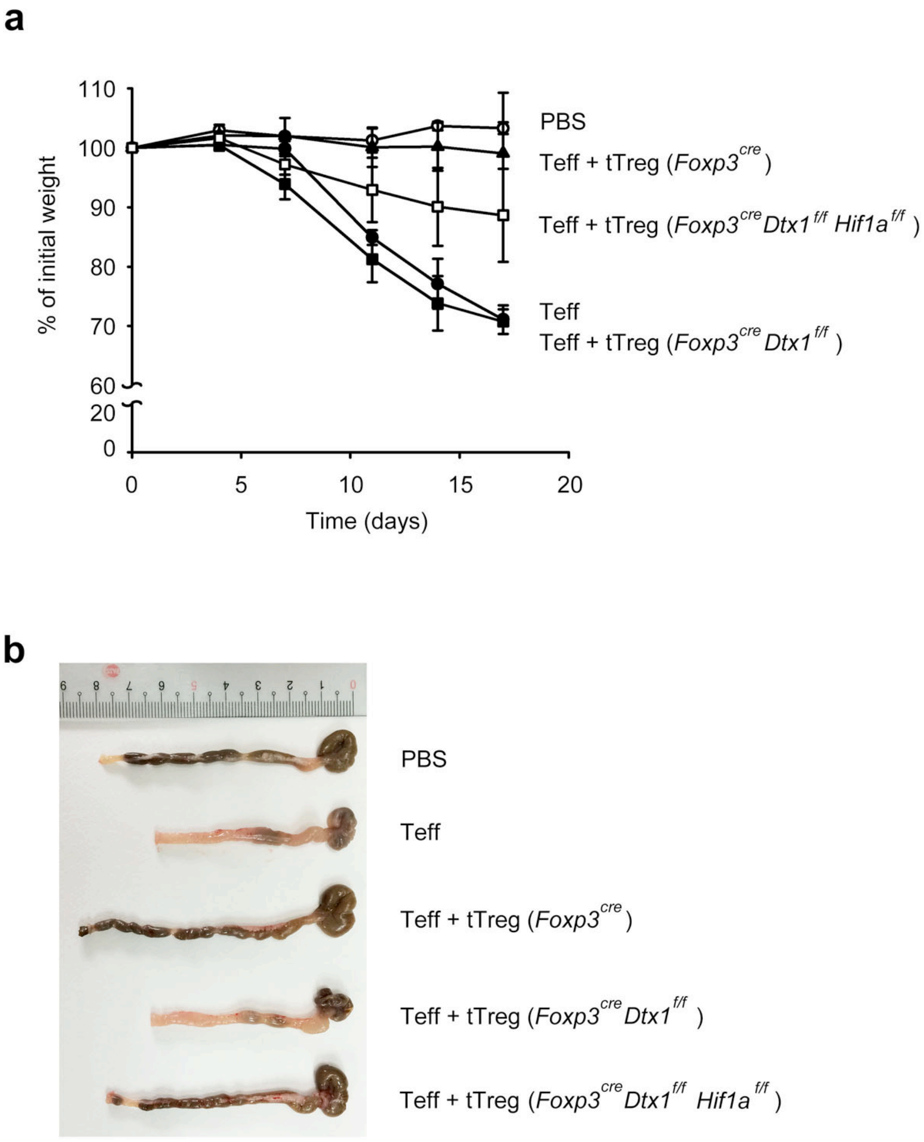

Supplementary Figure 12. HIF-1α-deficiency restores the ability of *Dtx1*$^{-/-}$ tTreg cells to inhibit colitis. $CD4^{+}CD25^{-}$ T cells (4×10^5) from female B6 mice or PBS were administered intraperitoneally into female *Rag1*$^{-/-}$ mice with or without 1×10^5 tTregs from female mice, as indicated. Body weight (a) was assessed from day 4 and intestines (b) were isolated at day 17 for morphological examination. n = 4.

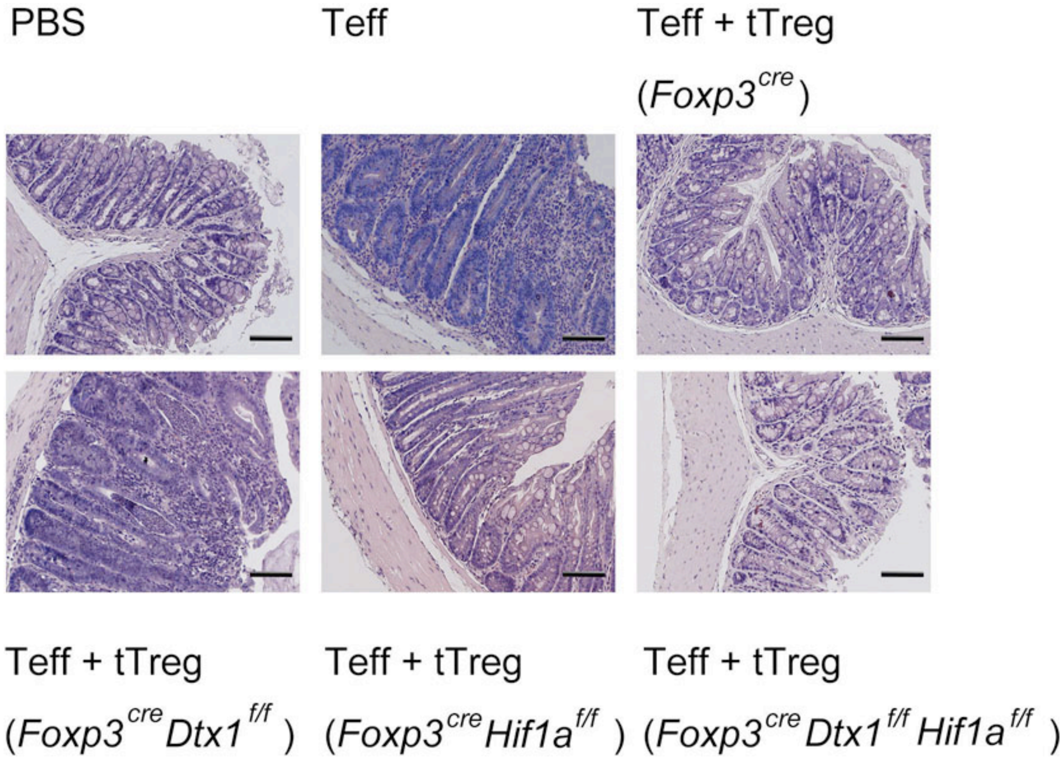

Supplementary Figure 13. *Dtx1$^{-/-}$Hif1a$^{-/-}$* tTregs efficiently inhibit effector T cell-induced colitis in *Rag1$^{-/-}$* mice. Mice in Fig. 6f were sacrificed and colons were removed, cleaned, fixed in paraformaldehyde, embedded in paraffin, sectioned, and stained with hematoxylin and eosin (H&E). Micrographs are representative of the 3 mice in each group. Bar indicates 100 μm.

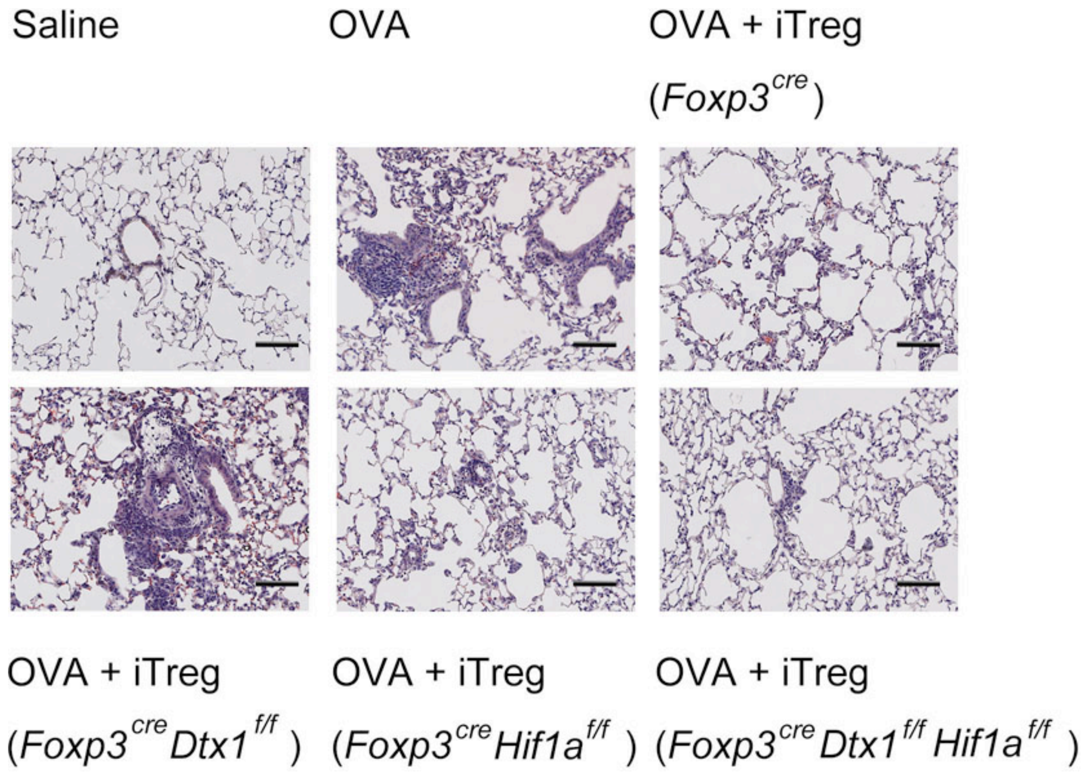

Supplementary Figure 14. HIF-1α-deficiency restores the ability of *Dtx1*$^{-/-}$ iTreg cells to inhibit airway hypersensitivity. Lung tissue was isolated from mice in Fig. 6g. Sections of lung were examined after staining with H&E. Micrographs are representative of the 3 mice in each group. Bar indicates 100 μm.

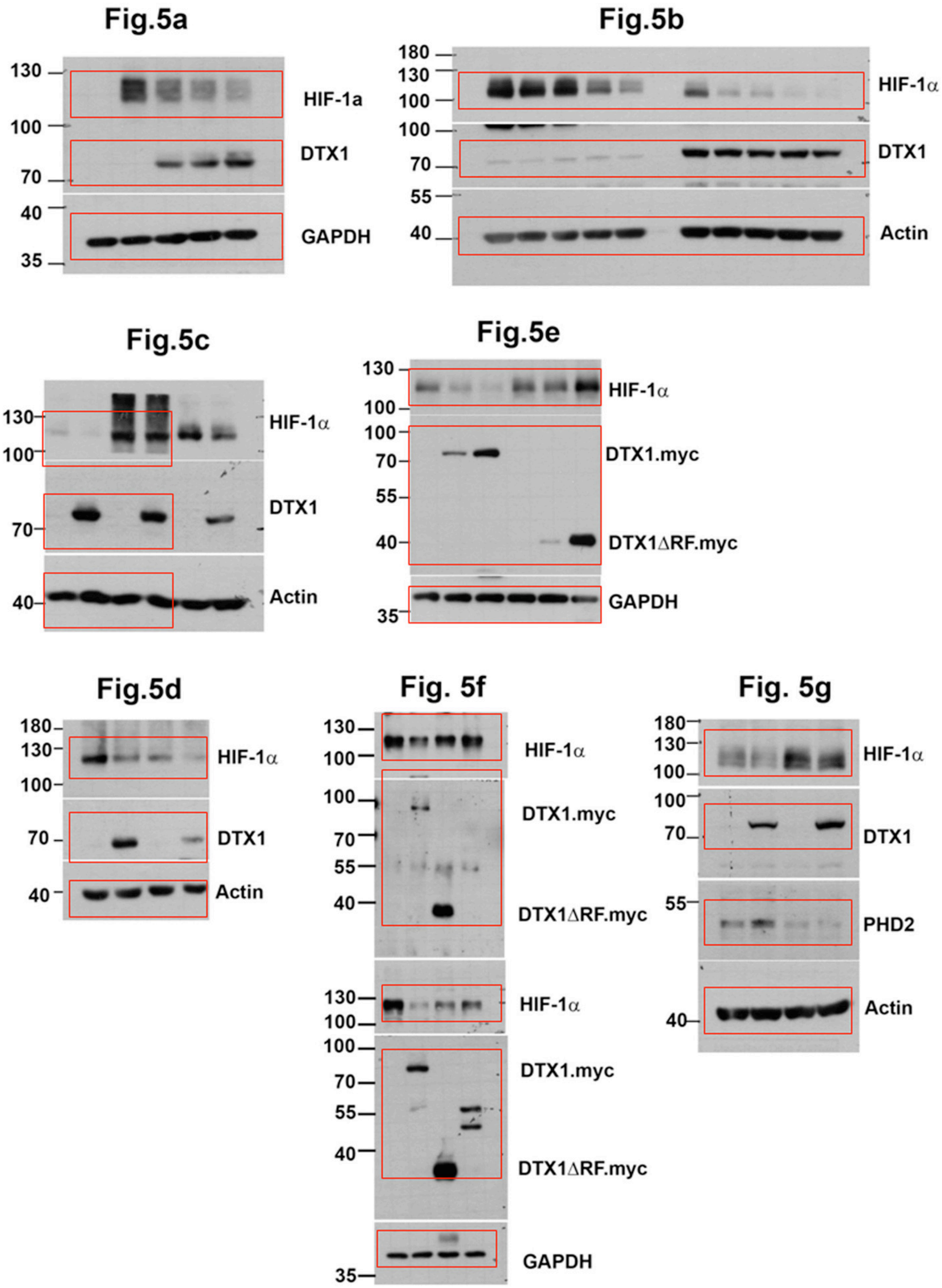

Supplementary Figure 15. Uncropped images of the original scans of immunoblots.
Uncropped, full-size scans of immunoblots shown in Fig. 5a -5g.

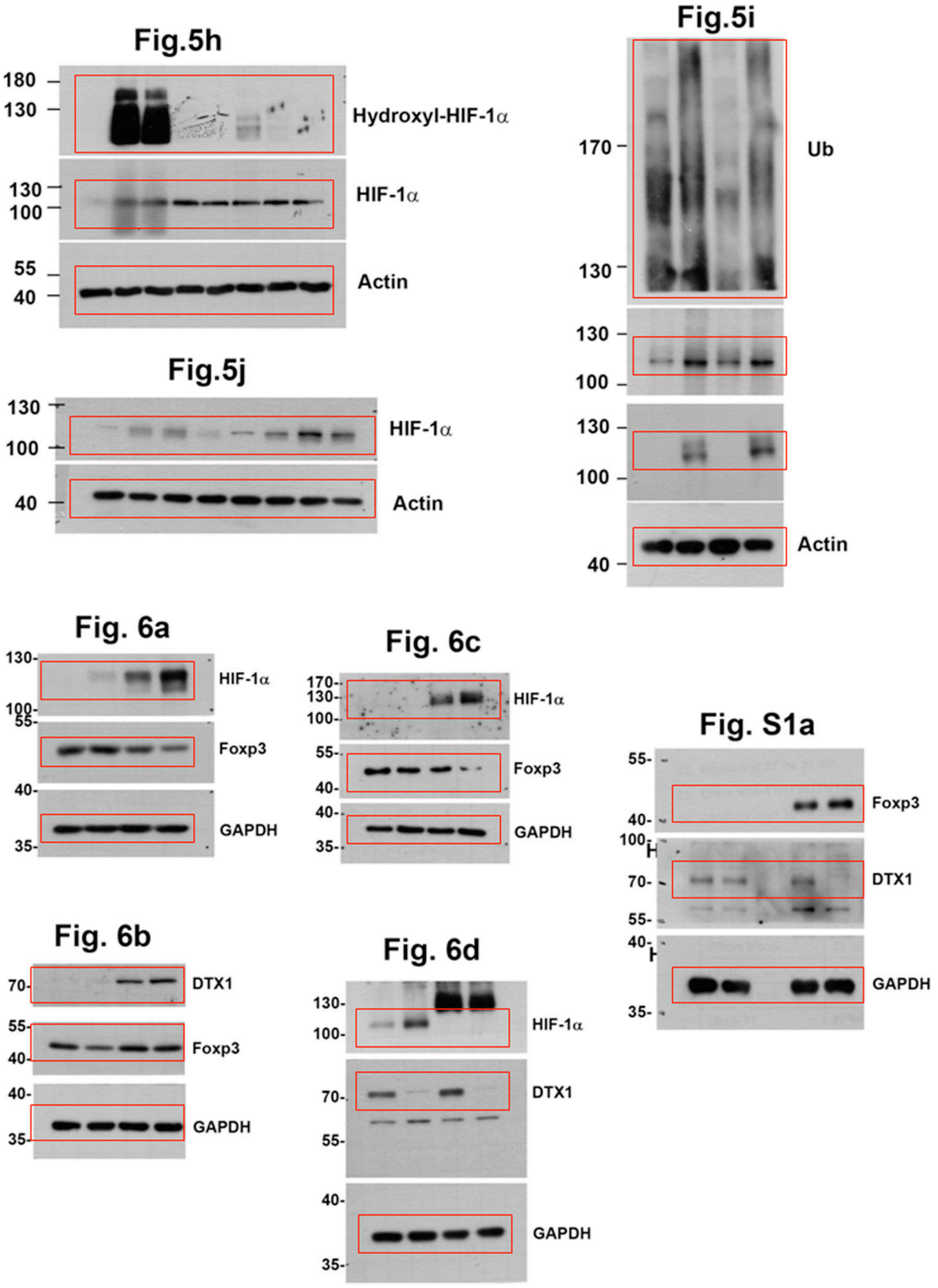

Supplementary Figure 16. Uncropped images of the original scans of immunoblots. Uncropped, full-size scans of immunoblots shown in Fig. 5h-5j, 6a-6d, and Supplementary Fig. 1a.

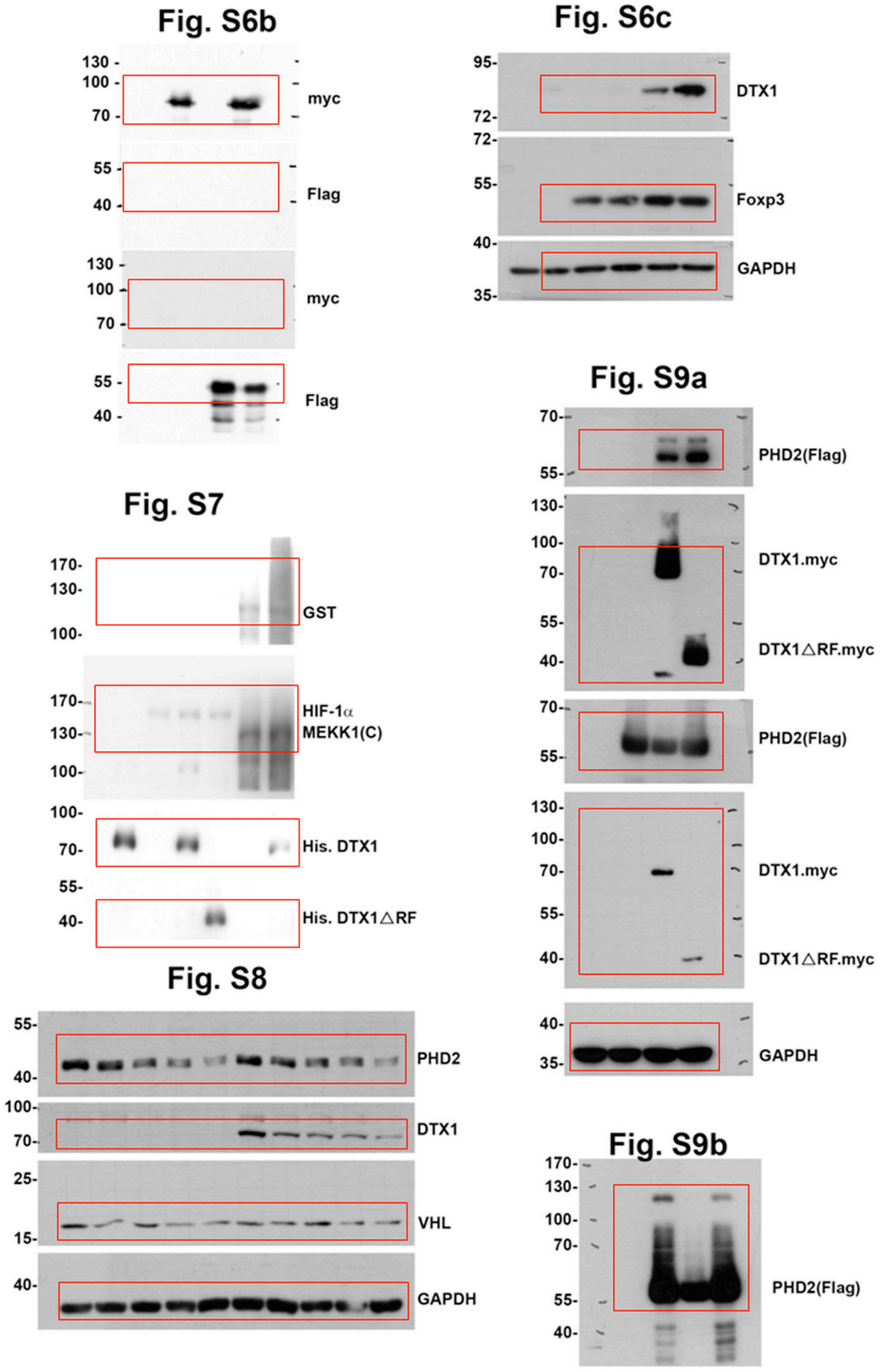

Supplementary Figure 17. Uncropped images of the original scans of immunoblots. Uncropped, full-size scans of immunoblots shown in Supplementary Fig. 6b, 6c, 7, 8, and 9.
